# Technology-Enabled (P)rehabilitation for Patients Undergoing Cancer Surgery: A Systematic Review and Meta-Analysis

**DOI:** 10.3390/cancers18020296

**Published:** 2026-01-18

**Authors:** Tiffany R. Tsoukalas, Zirong Bai, Claire Jeon, Roy Huynh, Eva Gu, Kate Alexander, Paula R. Beckenkamp, Adrian Boscolo, Kilian Brown, Phyllis Butow, Sharon Carey, Fang Chen, Meredith Cummins, Haryana M. Dhillon, Vesna Dragoje, Kailey Gorman, Matthew Halpin, Abby Haynes, Ilona Juraskova, Sascha Karunaratne, Jamie Keck, Bora Kim, Cherry Koh, Qiang Li, Lara Lipton, Xiaoqiu Liu, Jaime Macedo, Rebecca Mercieca-Bebber, Renee Moreton, Rachael L. Morton, Julie Redfern, Bernhard Riedel, Angus Ritchie, Charbel Sandroussi, Cathy Slattery, Allan Ben Smith, Michael Solomon, Flora Tao, Kate White, Kate Wilson, Kahlia Wolsley, Kun Yu, Daniel Steffens

**Affiliations:** 1 Faculty of Medicine and Health, Central Clinical School, The University of Sydney, Sydney, NSW 2050, Australia; tiffany.tsoukalas@health.nsw.gov.au (T.R.T.); zirong.bai@health.nsw.gov.au (Z.B.); jredfern@bond.edu.au (J.R.);; 2 Surgical Outcomes Research Centre (SOuRCe), Royal Prince Alfred Hospital, Camperdown, Sydney, NSW 2050, Australia; 3 Sydney School of Health Sciences, Faculty of Medicine and Health, The University of Sydney, Sydney, NSW 2050, Australia; 4 Sydney Local Health District, Sydney, NSW 2050, Australia; 5 Institute of Academic Surgery (IAS), Royal Prince Alfred Hospital, Sydney, NSW 2050, Australia; 6 Department of Colorectal Surgery, Royal Prince Alfred Hospital, Sydney, NSW 2050, Australia; 7 Centre for Medical Psychology and Evidence-Based Decision-Making (CeMPED), School of Psychology, Faculty of Science, The University of Sydney, Sydney, NSW 2050, Australia; 8 Psycho-Oncology Cooperative Research Group, School of Psychology, Faculty of Science, The University of Sydney, Sydney, NSW 2050, Australia; 9 School of Psychology, The University of Sydney, Sydney, NSW 2050, Australia; 10 The Data Science Institute, University of Technology Sydney, Sydney, NSW 2007, Australia; 11 NeuroEndocrine Cancer Australia, Blairgowrie, VIC 3942, Australia; 12 Sydney Health Care Interpreter Service, Sydney Local Health District, Sydney, NSW 2050, Australia; 13 Bowel Cancer Australia, Sydney, NSW 2060, Australia; kailey@bowelcanceraustralia.org; 14 Institute for Musculoskeletal Health, Sydney Local Health District, Sydney, NSW 2050, Australia; abby.haynes@sydney.edu.au; 15 School of Public Health, Faculty of Medicine and Health, The University of Sydney, Sydney, NSW 2050, Australia; 16 Department of Colorectal Surgery, St Vincent’s Private Hospital, Fitzroy, VIC 3065, Australia; 17 The Daffodil Centre, The University of Sydney and Cancer Council NSW, Sydney, NSW 2011, Australia; 18 The George Institute for Global Health, University of New South Wales, Sydney, NSW 2031, Australia; 19 Cabrini Health, Malvern, VIC 3144, Australia; 20 Rare Cancers Australia, Bowral, NSW 2576, Australia; 21 NHMRC Clinical Trials Centre, The University of Sydney, Sydney, NSW 2050, Australia; 22 Institute for Evidence-Based Healthcare, Bond University, Robina, QLD 4226, Australia; 23 Department of Anaesthesia, Perioperative Medicine, and Pain Medicine, Peter MacCallum Cancer Centre, Melbourne, VIC 3000, Australia; 24 The Sir Peter MacCallum Department of Oncology, The University of Melbourne, Melbourne, VIC 3000, Australia; 25 The Department of Critical Care, Critical Care, The University of Melbourne, Parkville, VIC 3010, Australia; 26 Health Informatics Unit, Sydney Local Health District, Camperdown, NSW 2050, Australia; 27 Concord Clinical School, Faculty of Medicine and Health, The University of Sydney, Sydney, NSW 2050, Australia

**Keywords:** oncology, abdominal surgery, thoracic surgery, digital health, prehabilitation, rehabilitation, patient-reported outcomes

## Abstract

**Background/Objectives:** (P)rehabilitation, comprising structured exercise, nutritional optimisation, and/or psychological support delivered pre- or postoperatively, has demonstrated efficacy in improving outcomes across the cancer care continuum. However, access remains limited. Technology-enabled (p)rehabilitation offers a novel solution with the potential to enhance equity and continuity of care. This systematic review aimed to evaluate the efficacy of technology-enabled (p)rehabilitation on perioperative and patient-reported outcomes among individuals undergoing thoracic and/or abdominopelvic cancer surgery. **Methods:** Six databases were search from inception to October 2024. Eligible studies were randomised controlled trials (RCTs) comparing technology-enabled (p)rehabilitation with usual care, placebo, or non-technology-based interventions in adults undergoing thoracic and/or abdominopelvic cancer surgery. Outcomes included postoperative complications, hospital readmissions, hospital length of stay (LOS), quality of life (QoL), pain, anxiety, depression, fatigue, distress, and satisfaction. Higher scores indicated improved QoL or worse symptom severity. Risk of bias was assessed using the revised Cochrane tool, and evidence strength was determined using GRADE methodology. Relative risks (RR) and mean differences (MD) were calculated using random-effects meta-analysis. **Results:** Seventeen RCTs (18 publications, n = 1690) were included. Trials most commonly evaluated application-based platforms (n = 8) and the majority exhibited some risk of bias. Technology-enabled (p)rehabilitation was associated with a significant reduction in LOS (MD = 1.33 days; 95% CI: 0.59–2.07; seven trials), and improvements in pain (MD = 6.12; 95% CI: 3.40–8.84; four trials), depression (MD = 2.82; 95% CI: 0.65–4.99; five trials), fatigue (MD = 10.10; 95% CI: 6.97–13.23; three trials) and distress (MD = 1.23; 95% CI: 0.30–2.16; single trial) compared with controls. **Conclusions:** Technology-enabled (p)rehabilitation shows promise in reducing LOS and improving selected patient-reported outcomes following thoracic and abdominopelvic cancer surgery. Although evidence is limited due to the small number of studies, modest sample sizes, methodological heterogeneity, and intervention variability, the overall findings justify further investigation. Large-scale, adequately powered clinical trials are required to confirm efficacy and guide clinical effectiveness and implementation studies.

## Simple Summary

Preoperative and postoperative programs that incorporate exercise, nutritional support, and/or psychological care, collectively known as (p)rehabilitation, have demonstrated efficacy in improving cancer patient outcomes. Access to these programs, however, remains limited. Technology-enabled (p)rehabilitation offers a potential solution to enhance equity and continuity of care. This review evaluated the impact of technology-enabled (p)rehabilitation on perioperative and patient-reported outcomes in individuals undergoing thoracic and/or abdominopelvic cancer surgery. Seventeen randomised controlled trials, involving 1690 participants, were analysed. Findings suggest that technology-enabled (p)rehabilitation significantly reduces hospital length of stay, and improves patient reported outcomes such as pain, depression, fatigue, and distress compared with control groups. Despite these encouraging results, the evidence is limited by small sample sizes and methodological variability. Large-scale clinical trials are needed to confirm efficacy and inform implementation strategies.

## 1. Introduction

Cancer is the leading cause of death worldwide, accounting for nearly 10 million deaths in 2020 [1,2]. By 2050, its global burden is expected to rise substantially, with annual diagnosis and mortality exceeding 35 million and 18 million respectively [1,3,4].

For patients with localised disease, surgical resection, with or without adjuvant therapy, remains the cornerstone of curative treatment. However, major oncologic surgery imposes a significant physiological and psychological burden on patients. Physiologically, surgery triggers inflammatory and catabolic responses that exacerbate sarcopenia and functional decline. Psychologically, patients may experience anxiety, depression and/or emotional distress related to diagnostic uncertainty, symptom burden and prognostic concerns. Even with modern perioperative strategies, these burdens predispose patients to postoperative complications, prolonged hospitalisation, delayed functional recovery, and increased risk of hospital readmission. Collectively, these sequelae contribute to cycles of deconditioning, malnutrition, and psychological distress, adversely impacting patient outcomes, healthcare utilisation, timely access to postoperative adjuvant therapy, and long-term survivorship.

Structured prehabilitation and rehabilitation programs represent key strategies to support surgical recovery. Prehabilitation is a preoperative intervention aimed at optimising patients’ resilience, while rehabilitation is a postoperative intervention designed to support recovery and restore functional capacity. Such interventions are typically delivered as comprehensive programs combining exercise, nutritional optimisation, and psychological support. Conventional face-to-face (p)rehabilitation programs have demonstrated efficacy in improving surgical and functional outcomes across the cancer care continuum [5,6,7]. However, their implementation is often constrained by logistical barriers, including limited availability of specialised services, scheduling challenges, and geographic inaccessibility [8,9,10], which limit scalability and exacerbate inequities in access and uptake.

Technology-enabled (p)rehabilitation has emerged as a promising, scalable approach to address these implementation barriers [11]. These programs leverage digital health platforms, including synchronous telehealth consultations, asynchronous mobile applications, wearable activity monitors and biosensors, and immersive virtual reality environments, to deliver structured, multimodal care across the surgical treatment pathway. Key distinguishing features include real-time remote monitoring of physiological parameters and activity levels, automated delivery of educational content and exercise prescriptions, bidirectional communication between patients and healthcare providers, and adaptive program tailoring based on individual progress and feedback. By decentralising care delivery, technology-enabled interventions have the potential to overcome geographic barriers, reduce travel burden, enhance program adherence through convenience and personalisation, and enable continuous patient engagement throughout the perioperative period. Despite growing clinical interest, evidence for the efficacy of such programs is limited, with existing reviews primarily examining conventional, non-technology based programs [6,12] or broad cancer cohorts [6,13,14].

To address this, we conducted a systematic review and meta-analysis using the PICO framework. The population included adults undergoing elective thoracic and/or abdominopelvic cancer surgery. The intervention comprised technology-enabled prehabilitation and/or rehabilitation programs. The comparator was standard care, usual care, or non-technology-enabled (p)rehabilitation interventions. The outcomes assessed included postoperative complications, hospital length of stay, readmission rates, patient-reported outcome measures (PROMs), and patient-reported experience measures (PREMs).

## 2. Methods

This systematic review was conducted and reported in accordance with the Preferred Reporting Items for Systematic Reviews and Meta-Analysis (PRISMA) Statement [15] (Supplementary Table S1). The protocol was prospectively registered on PROSPERO (CRD42024601602).

### 2.1. Search Strategy

The search strategy was developed in consultation with a senior University of Sydney Librarian. Six electronic databases (PubMed, MEDLINE/EMBASE, Web of Science, CENTRAL, and CINAHL) were searched from database inception to October 2024. Forward and backward citation tracking was also performed to identify any additional eligible studies. The complete search strategy is provided in Supplementary Table S2.

### 2.2. Eligibility Criteria

The eligibility criteria was defined using the Population, Intervention, Comparator, Outcomes, and Study Design (PICOS) framework [16]. The target population comprised adults (≥18 years) undergoing thoracic and/or abdominopelvic cancer surgery. Interventions of interest included technology-enabled (p)rehabilitation programs. Interventions could be unimodal (exercise, nutrition, or psychosocial training/support) or multimodal (any combination). Interventions were considered technology-enabled if delivered via mobile application, web-based platform, video game, or virtual reality. For the purpose of this study (p)rehabilitation programs included interventions delivered prior to surgery (prehabilitation), and/or interventions initiated within 30 days post-index surgery (rehabilitation). No restrictions were placed on program duration or location. Eligible comparators included no intervention, placebo, minimal intervention, or non-technology-based controls. Outcomes of interest were postoperative complications, hospital readmission rates, hospital length of stay, health-related quality of life, pain, anxiety, depression, fatigue, distress, patient satisfaction. Only randomised controlled trials (RCTs) were eligible. Trials reported solely as scientific conference abstracts were excluded. No restrictions were applied on language or publication date.

### 2.3. Study Selection

All retrieved publications were imported into Covidence for deduplication and screening. Two reviewers independently screened titles and abstracts, followed by full-text review. Discrepancies were resolved through discussion, with a third reviewer consulted if consensus could not be reached.

### 2.4. Data Extraction

A piloted extraction form was used to record study characteristics (publication year, sample characteristics, target population), intervention and comparator details, and outcome measures. Where multiple publications of the same trial existed, data was consolidated to maximise completeness. Where multiple intervention groups were included, only the technology-enabled (p)rehabilitation intervention group and the control group, were included in the analysis. Non-technology-enabled intervention groups were excluded [17].

For dichotomous outcomes (e.g., postoperative complications, hospital readmissions), the number of events and participants per arm were extracted. For continuous outcomes (e.g., hospital length of stay, patient-reported outcome measures), means and standard deviations were preferentially extracted, along with the number of patients analysed in each arm.

Where medians and ranges (interquartile or minimum-maximum) were reported, data were converted to means and standard deviations using the method of Wan et al. [18]. Other data formats were transformed as necessary [17,19]. Patient-reported outcome measures were scaled to 0–100 (when required). Details of outcome transformation and standardisation are provided in Supplementary Table S3. Higher scores indicated better health-related quality of life or worse symptom severity (pain, anxiety, depression, fatigue, distress).

Data presented solely in figures were estimated to two decimal places using WebPlotDigitizer (version 5.2). Studies that reported outcomes using multivariable models (e.g., Generalised Estimating Equations) and/or presented between-group differences without effect size estimates, were excluded from pooled analysis. Excluded outcomes are detailed in Supplementary Table S4.

Outcome data were categorised into six predefined timepoints: baseline (prior to intervention), preoperative (prior to surgery), immediate postoperative (surgery day—postoperative day seven), early postoperative (one week–one month), intermediate postoperative (one–three months), and long-term postoperative (>three months) (Supplementary Table S5A,B). When multiple tools assessed the same outcome at a given timepoint, or when multiple assessments occurred within the same predefined timepoint, a prespecified prioritisation hierarchy was applied (Supplementary Table S6A,B).

### 2.5. Risk of Bias and Certainty of Evidence

Two reviewers (TT, ZB) independently assessed the risk of bias of included studies using Version 2 of the Cochrane Risk-of-Bias Tool for Randomised Trials (RoB2) [20]. Bias was assessed across five domains (randomisation process, deviations from intended interventions, missing outcome data, measurement of the outcome, and selection of the reported results), and trials were classified as “low”, “some concerns”, or “high” risk of bias. Disagreements were resolved by discussion, with a third reviewer consulted if consensus could not be reached.

The quality and certainty of the evidence was rated accordingly to the Grading of Recommendations, Assessment, Development, and Evaluations (GRADE) [21]. Evidence was downgraded by one level according to the following criteria: (1) risk of bias (classification of one or more domain as ‘high risk’ in ≥25% of included trials); (2) inconsistency (statistically significant heterogeneity [I^2^ > 50%] or ≤75% of trials reporting results in the same direction); (3) imprecision (sample size <300 participants for dichotomous outcomes or <400 for continuous outcomes); and (4) publication bias (identified by visual assessment of funnel plots if >10 trials were included) [12]. The indirectness criterion was not considered since all studies involved thoracic and/or abdominopelvic populations, with direct comparisons and relevant outcomes. For single trials with <400 participants, inconsistency and imprecision (i.e., sparse data) were both downgraded, and the quality of evidence was rated as ‘low’ [12]. If additional risk of bias limitation were identified, the quality of the evidence could be further downgraded to ‘very low’ [12].

### 2.6. Data Synthesis and Analysis

All analyses were conducted using the Comprehensive Meta-Analysis software (version 4). A random effects model was applied. For dichotomous outcomes, pooled relative risks (RRs) and 95% confidence intervals (CI) were calculated. An RR < 1 indicated benefit of the intervention. For continuous outcomes, pooled mean differences (MDs) with 95% CIs were calculated. Mean differences were coded so that positive values favoured (p)rehabilitation interventions, with scores inverted for outcomes where higher values represent improved outcomes (i.e., quality of life). Statistical heterogeneity was assessed using the χ^2^ test (*p* < 0.10) and quantified with the I^2^ statistic, with I^2^ > 50% considered substantial heterogeneity. Where meta-analysis was not appropriate, results were reported descriptively.

## 3. Results

**Figure 1:**
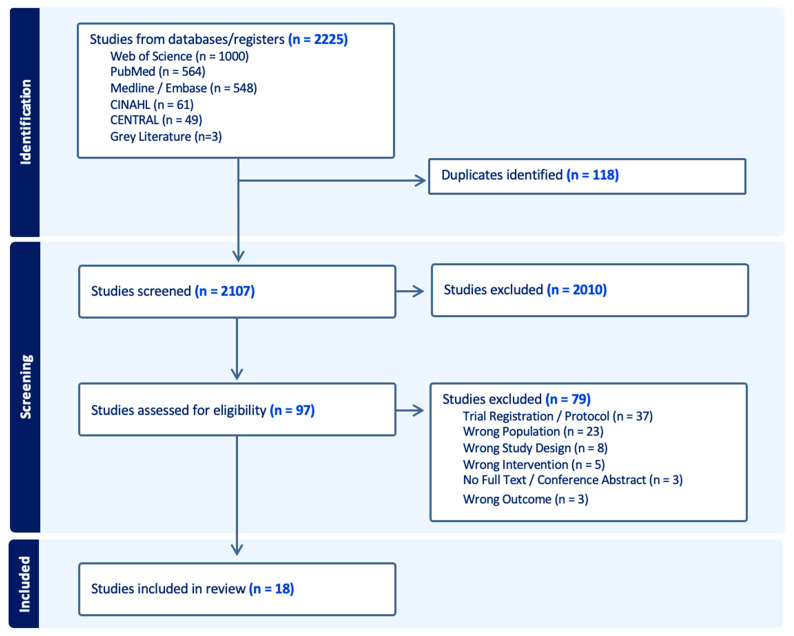
PRISMA (Preferred Reporting Items for Systematic reviews and Meta-Analyses) Flow Chart.

Of the 2225 publications identified, a total of 2107 were screened, with 97 undergoing full-text review. Of these, 18 publications (reporting findings from 17 unique trials) met the eligibility criteria (Figure 1). The primary reason for full-text exclusion is detailed in Supplementary Table S7.

### 3.1. Characteristics of Included Publications

Of the 17 included trials (n = 1690 participants), eight evaluated prehabilitation interventions exclusively [22,23,24,25,26,27,28,29], five focused solely on rehabilitation [30,31,32,33,34,35], and four incorporated both prehabilitation and rehabilitation components [36,37,38,39]. The majority of trials included patients undergoing surgery for gastrointestinal cancer. Of these, five focused on upper gastrointestinal cancer (n = 567 participants) [25,27,30,31,35,37], five on lower gastrointestinal cancer (n = 328 participants) [24,28,29,34,38], and one on metastatic gastrointestinal cancer broadly (n = 26 participants) [36]. This was followed by thoracic cancer (three trials; n = 431 participants) [23,33,39], genitourinary cancer (single trial; n = 203 participants) [22], gynaecological cancer (single trial; n = 67 participants) [26], and hepatobiliary cancer (single trial; n = 68 participants) [32]. Sample sizes ranged from 22 to 203. Detailed information of the included trials is included in Table 1, Table 2 and Table 3.

Intervention delivery modalities varied across the included trials. Application-based platforms were most frequent (n = 8), followed by virtual reality (n = 4). Web-based platforms, telehealth, videogame-based, wearable technology, and a multimedia video were each used in a single study. Across all trials, 10 incorporated psychological support, 10 physical activity, and 7 nutritional support.

### 3.2. Risk of Bias

**Figure 2:**
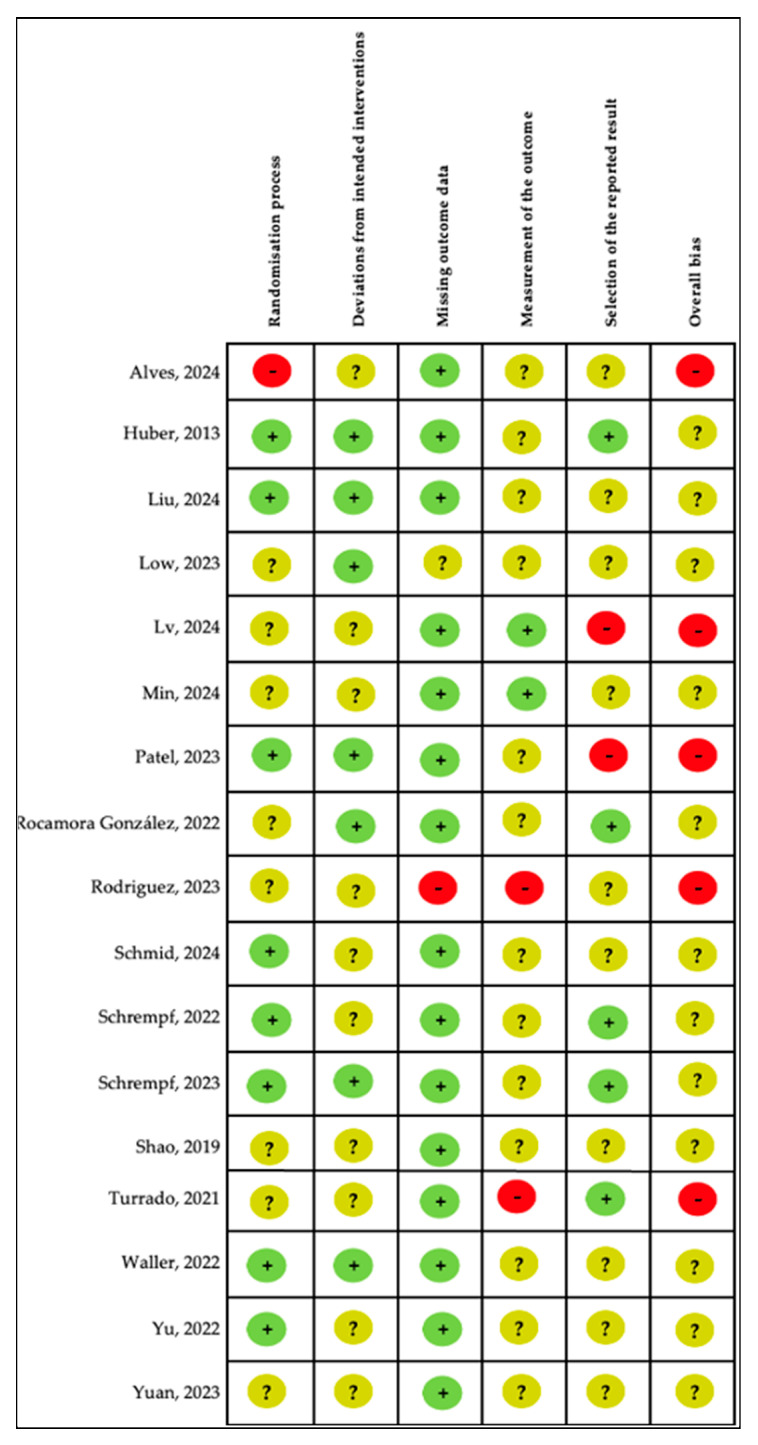
Risk of bias using Version 2 of the Cochrane Risk-of-Bais Tool for Randomised Trials (RoB2). Green “+” indicates low risk of bias; yellow “?” indicates some concerns; and red “-” indicates high risk [22,23,24,25,26,27,28,29,30,31,32,33,34,35,36,37,38,39].

The risk of bias assessment for the included trials is presented in Figure 2. Most trials presented some risk of bias. Bias due to ‘measurement of the outcomes’ and ‘selection of the reported result’ were most frequently judged to be at high risk, while ‘deviations from intended interventions’ was least commonly rated as a high risk of bias.

### 3.3. Certainty of Evidence

The quality and certainty of the evidence, according to the Grading of Recommendations, Assessment, Development, and Evaluations, is presented in Table 4.

### 3.4. Efficacy of (P)rehabilitation Programs

#### 3.4.1. Postoperative Complications

Six trials (n = 552 participants) evaluated the impact of technology-enabled (p)rehabilitation on postoperative complications. Intervention timing varied: two trials focused on rehabilitation only, two on prehabilitation only and two combined both. Intervention components were heterogenous: two trials delivered multimodal programs incorporating physical activity, nutritional support and psychological support; two delivered a unimodal exercise program; and two delivered psychological support.

Reporting methods varied: three trials (50%) used the Clavien–Dindo classification system [33,34,38], two (33%) reported complications without formal grading [28,37], and one (17%) used the Modified Accordion Grading System [25]. Three trials solely reported ‘major complications’, defined as either Clavien–Dindo Grade ≥III (n = 2) [34,38] or ≥IIIb (n = 1) [33]. Additional instruments were reported in the included trials, but these were not included in the pooled analysis (Table 2 and Table 3).

Four trials (67%) [28,33,34,37] reported equivalent or reduced postoperative complication events in the intervention group, compared to the control group. However, pooled analysis found no statistically significant difference between groups (RR = 0.95; 95% CI = 0.69 to 1.32; I2 = 0) (Supplementary Figure S1). The quality of evidence for this outcome was rated as low.

#### 3.4.2. Hospital Readmission

Two trials (n = 162 participants) [33,36] investigated the effect of (p)rehabilitation interventions on 30-day hospital readmission rates. One trial delivered rehabilitation as a multimodal program incorporating physical activity, nutritional support and psychological support, while the other evaluated a combined prehabilitation and rehabilitation program focused on physical activity. Pooled analysis demonstrated no statistically significant difference between intervention and control groups (RR = 1.46; 95% CI = 0.57 to 3.76; I2 = 0) (Supplementary Figure S1). The quality of evidence for this outcome was rated as low.

#### 3.4.3. Hospital Length of Stay

The efficacy of technology-enabled (p)rehabilitation on hospital length of stay was reported across seven trials (n = 707 participants) [23,28,30,31,34,37,38,39]. Intervention timing varied: three combined both prehabilitation and rehabilitation, two focused on rehabilitation, and two on prehabilitation. Intervention components were heterogenous: two trials delivered a unimodal exercise program, two provided psychological support, two combined physical activity with nutritional support, and one delivered a multimodal program incorporating physical activity, nutritional support and psychological support.

Pooled estimates demonstrated moderate quality evidence of a significant effect favouring (p)rehabilitation over standard care (MD = 1.33 days; 95% CI = 0.59 to 2.07; I^2^ = 4.1) (Supplementary Figure S2).

#### 3.4.4. Health-Related Quality of Life (QoL)

Six trials (n = 501 participants) evaluated the effect of technology-enabled (p)rehabilitation on health-related quality of life. Intervention timing varied: three trials evaluated rehabilitation only, two prehabilitation only and one combined both. Intervention components ranged from unimodal programs targeting physical activity or psychological support, to combined interventions integrating nutritional support with either physical activity or psychological support.

Reporting methods varied, with each trial using a different measure. These included the European Organisation for Research and Treatment of Cancer Quality of Life Questionnaire—Core 30 (EORTC QLQ-C30; Global Health Status [32] and Summary Score [35]), EuroQol 5-Dimension 5-Level (EQ-5D-5L; Overall Health Component [23] and Index Score [34]), World Health Organisation Quality of Life (WHO QLQ; Physical Health component [24]), and the Functional Assessment of Cancer Therapy [36] (FACT). Scores were standardised to a 0–100 range to enable comparability across measures (Supplementary Table S3). Additional instruments were reported in the included trials, but these were not included in the pooled analysis (Table 2 and Supplementary Table S4).

Overall pooled analysis demonstrated no significant effect between the control and intervention groups (MD = −0.05; 95% CI = −0.81 to 0.72). When stratified by timepoint, a significant improvement favouring (p)rehabilitation was observed at intermediate (single trial [32]; MD = 7.50; 95% CI = 0.65 to 14.35) and long-term follow-up (n = 2 trials [32,35]; MD = 9.93; 95% CI = 4.34 to 15.51) (Supplementary Figure S3).

#### 3.4.5. Pain

Four randomised controlled trials (n = 469 participants) [23,30,33,35] evaluated the effect of technology-enabled (p)rehabilitation on postoperative pain. Three trials focused on rehabilitation, and one on prehabilitation. Across these studies, intervention components ranged from unimodal programs targeting physical activity, to combined strategies integrating nutritional support with either physical activity or psychological support. One trial delivered a multimodal program including physical activity, nutritional support and psychological support.

Reporting methods also varied, with each trial using a different measure. These included the Numeric Rating Scale, EORTC QLQ-C30 pain subscale, MD Anderson Symptom Inventory for Lung Cancer pain score, and the EQ-5D-5L pain/discomfort component. Scores were standardised to a 0–100 range to enable comparability across measures (Supplementary Table S3). Additional instruments were reported in the included trials, but these were not included in the pooled analysis (Supplementary Table S4).

Overall pooled estimates demonstrated a significant difference between (p)rehabilitation and control groups (MD = 6.12, 95% CI = 3.40 to 8.84). When stratified by timepoint, a significant improvement favouring (p)rehabilitation was observed at immediate (n = 3 trials [23,30,33]; MD = 12.18, 95% CI = 7.19 to 17.17) and long-term follow-up (single trial [35]; MD = 8.10, 95% CI = 2.71 to 13.49). No data was available for preoperative or intermediate postoperative periods (Supplementary Figure S4).

#### 3.4.6. Anxiety

The effect of technology-enabled (p)rehabilitation on anxiety was evaluated in seven trials (n = 640 participants) [22,24,26,27,29,30,32]. Most trials focused on prehabilitation (n = 5), with two evaluating rehabilitation. Intervention components varied: unimodal programs targeted psychological support (n = 4 trials) or physical activity (single trial), while a combined program integrated physical activity with nutritional support (single trial), and a multimodal program included physical, nutritional and psychological components (single trial).

Reporting methods also varied: four trials (n = 242 participants) used the anxiety subscale of the Hospital Anxiety and Depression Scale (HADS-A), two (n = 331 participants) used the Spielberger State-Trait Anxiety Inventory (STAI) questionnaire, and one (n = 67 participants) used a novel six-tier Visual Facial Anxiety Scale. Scores were standardised to a 0–100 range to enable comparability across measures (Supplementary Table S3). Additional instruments were reported in the included trials, but these were not included in the pooled analysis (Table 1 and Table 2 and Supplementary Table S4).

Pooled estimates demonstrated no significant difference in anxiety between (p)rehabilitation and control groups (MD = 2.19; 95% CI = −0.08 to 4.46). Timepoint-stratified analysis indicated statistically significant reductions in anxiety at intermediate (single trial [32]; MD = 5.20; 95% CI = 0.22 to 10.18) and long-term (single trial [32]; MD = 7.62; 95% CI = 2.64 to 12.60) postoperative assessments (Supplementary Figure S5).

#### 3.4.7. Depression

Five randomised controlled trials (n = 268) [24,29,30,32,36] assessed the impact of technology-enabled (p)rehabilitation on depression. Intervention timing varied: two trials evaluated rehabilitation only, two prehabilitation only and one combined both. Intervention components were heterogenous, comprising unimodal programs targeting either physical activity (n = 2 trials) or psychological support (n = 2 trials). One trial implemented a multimodal program including physical, nutritional and psychological components.

Four trials (n = 242) used the depression subscale of the Hospital Anxiety and Depression Scale (HADS-D), and one (n = 26) used the Centre for Epidemiological Studies Depression Scale (CES-D). Scores were standardised to a 0–100 range to enable comparability across measures (Supplementary Table S3). Additional instruments were reported in the included trials, but these were not included in the pooled analysis (Table 2 and Supplementary Table S4).

**Figure 3:**
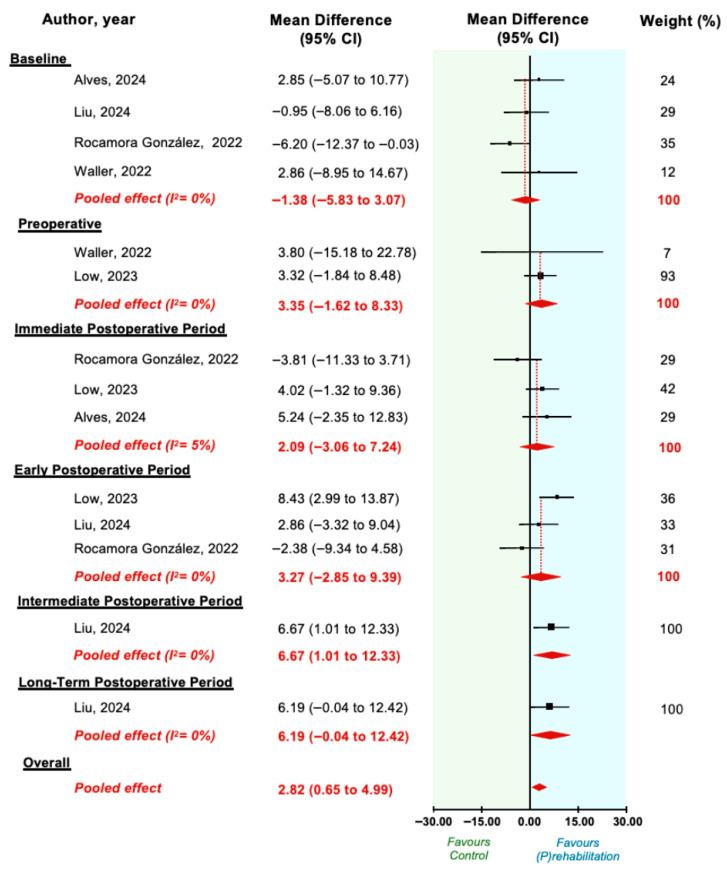
Mean difference in depression in randomised controlled trials of technology-enabled (p)rehabilitation for patients undergoing thoracic and/or abdominopelvic cancer surgery. (P)rehabilitation programs included interventions delivered prior to surgery (prehabilitation), and/or interventions initiated within 30 days post index surgery (rehabilitation). No restrictions were placed on program duration or location. Eligible comparators included no intervention, placebo, minimal intervention, or non-technology-based controls. Positive values favour prehabilitation interventions [24,29,30,32,36].

Pooled analysis showed a statistically significant reduction in depression favouring (p)rehabilitation (MD = 2.82; 95% CI = 0.65 to 4.99). Timepoint-stratified analyses revealed no significant between-group differences at baseline, preoperative, immediate, early, or long-term postoperative timepoints. A statistically significant reduction was observed at the intermediate postoperative timepoint (single trial [32]; MD = 6.67; 95% CI = 1.01 to 12.33) (Figure 3).

#### 3.4.8. Fatigue

Three trials (n = 374 participants) [30,33,35] evaluated the efficacy of technology-enabled (p)rehabilitation on fatigue. All trials delivered a rehabilitation program: one was a unimodal program targeting physical activity, another was a combined program integrating nutritional and psychological support, and the third was a multimodal program encompassing physical, nutritional, and psychological components.

Across included trials, reporting instruments included the Fatigue Assessment Scale, MD Anderson Symptom Inventory for Lung Cancer fatigue score and the EORTC QLQ-C30 fatigue subscale. Scores were standardised to a 0–100 range to enable comparability across measures (Supplementary Table S3). Additional instruments were reported in the included trials, but these were not included in the pooled analysis (Supplementary Table S4).

Pooled estimates demonstrated a significant reduction in fatigue favouring (p)rehabilitation over standard care (MD = 10.10; 95% CI = 6.97 to 13.23). Timepoint-stratified analyses revealed no significant between-group differences at baseline, immediate, and early postoperative periods. A statistically significant reduction in fatigue favouring (p)rehabilitation was reported at the long-term follow-up (single trial [35]; MD = 18.57; 95% CI = 13.77 to 23.37). No data were reported for the preoperative or intermediate postoperative periods (Supplementary Figure S6).

#### 3.4.9. Distress

The efficacy of technology-enabled (p)rehabilitation on distress was evaluated in a single trial (n = 200 participants) [39], using the Huaxi Emotional-Distress Index (scored on a 0–36 scale). The trial evaluated a combined prehabilitation and rehabilitation program incorporating both physical activity and nutritional support. Statistically significant differences were reported (MD = 1.23; 95% CI = 0.30 to 2.16) (Supplementary Figure S7).

#### 3.4.10. Patient Satisfaction

Nine of the included trials (53%) reported patient satisfaction, most commonly using brief self-reported questionnaires. Across intervention groups, satisfaction was consistently high. Five studies reported mean satisfaction scores ≥ 85% [22,24,35,36,39], one reported that 95.5% of participants rated their overall satisfaction as ≥3 on a five-point scale [33], and another reported that all participants rated the overall program as “good” or “excellent” [29]. No studies reported low satisfaction (Supplementary Table S8).

## 4. Discussion

This review synthesised the current evidence base on digital (p)rehabilitation in thoracic and abdominopelvic surgical oncology. Pooled analyses identified statistically significant reduction in length of hospital stay, pain, depression, fatigue and distress, but no consistent improvements were observed for postoperative complications, hospital readmissions, health-related quality of life, or anxiety. Timepoint-stratified analyses suggested improvements in health-related quality of life, pain, anxiety, depression, and fatigue mainly at later follow-up timepoints (>one month postoperatively), although these findings were largely derived from single trials.

In the present review, the absence of consistent effects on postoperative complications likely reflects the complex interplay between intervention timing and mechanistic pathways. Current evidence suggests that prehabilitation may reduce perioperative and in-hospital complications [6,11,40,41,42,43], whereas rehabilitation is primarily associated with improvements in recovery beyond the immediate postoperative period [44,45]. Aggregating prehabilitation and rehabilitation interventions under a single “(p)rehabilitation” framework may therefore obscure clinically meaningful effects. Findings from this review, particularly regarding postoperative complications, should therefore be interpreted with caution.

Findings in the present review align with prior evidence indicating that digital health interventions can enhance psychosocial outcomes. Telehealth programs for breast cancer survivors, for instance, have been associated with improved quality of life, reduced depression, lower distress, and perceived stress [46]. Similarly, digital pulmonary rehabilitation programs for lung cancer survivors have been associated with improvements in depression and anxiety [47], and digital prehabilitation programs for esophagogastric cancer cohorts have been associated with improvements in anxiety and emotional wellbeing [48]. As intervention modality is a key determinant of success [14], the accessibility and continuity of support inherent in digital programs likely underpin the observed benefits. However, interpretation of current findings is limited by the modest sample sizes and heterogeneity of included interventions. Findings from this review should therefore be interpreted with caution and further well-designed, adequately powered trials are needed to establish the most effective digital (p)rehabilitation models.

Previous systematic reviews in surgical oncology cohorts have primarily focused on conventional multimodal (p)rehabilitation (i.e., without the routine use of digital technology), with reported clinically meaningful improvements in functional recovery and reductions in complications. Notably, a large network meta-analysis by McIsaac et al. demonstrated significant benefits of multimodal prehabilitation [6]. Similarly, previous reviews of structured, in-person programs in lung cancer cohorts have reported reductions in major complications [12]. By contrast, the present review identified limited effect of (p)rehabilitation on clinical endpoints, with only a statistically significant improvement reported for length of hospital stay. This discrepancy may reflect the inherent constraints of digital interventions, particularly their inability to address postoperative complications that require in-person clinical assessment or intervention [49]. However, given the low overall certainty of evidence and high risk of bias across many included trials, further high-quality studies are needed to determine whether digital interventions can achieve comparable effects to conventional, in-person models.

Improvements in functional and exercise capacity in the present review were predominately observed at intermediate and long-term follow-up. This temporal pattern is consistent with findings from a recent meta-analysis which reported statistically significant improvements in exercise capacity at four to eight weeks postoperatively among patients receiving prehabilitation, compared with usual care [50]. Consistent with this pattern, a pilot study by Van Rooijen et al. reported that at four weeks postoperatively, 86% of patients in the prehabilitation group had returned to or exceeded their baseline functional capacity, compared with 40% of controls [51]. Collectively, these findings underscore the importance of assessing outcomes beyond the immediate postoperative period and suggest that future research should explore the optimal timing and duration of digital interventions to maximise recovery.

Despite growing evidence supporting (p)rehabilitation interventions, interpreting trial findings remains challenging due to variability in design, reporting, and outcome assessment. A persistent issue is the lack of standardisation in intervention protocols and outcome measures [11,52,53,54,55]; methodological limitations contributing to the substantial heterogeneity observed in the present review. Further complicating analysis, patient-reported outcome measures (PROMs) and patient-reported experience measures (PREMs), which are increasingly valued for capturing recovery trajectories and long-term well-being, are often underappreciated in traditional grading frameworks [56]. Such frameworks tend to prioritise objective or blinded measures, inadvertently penalising patient-centred research. As a result, studies using PROMs and PREMs were often rated as “some concerns” or “high risk” for outcome measurement, despite their established prognostic relevance in oncology research. Future studies should therefore focus on improving methodological rigour while refining grading systems to ensure that clinically meaningful outcomes, including PROMs and PREMs, are appropriately recognised and appraised.

### 4.1. Strengths and Limitations

This review has several methodological strengths. The protocol was pre-registered, and the review adhered to Cochrane Handbook standards. A comprehensive search strategy—developed in conjunction with a senior librarian at the University of Sydney—was applied without language restrictions. Inclusion was limited to randomised controlled trials, and risk of bias and certainty of evidence were assessed using RoB 2 and GRADE, respectively.

Nonetheless, several limitations that may affect the robustness and generalisability of the findings warrant consideration. First, the relatively small number of trials, modest sample sizes and predominance of gastrointestinal cancer trials (67%) limited statistical power and generalisability of the findings. Specifically, the small number of eligible studies precluded subgroup analyses stratified by intervention timing, modality or components, limiting the ability to discern conclusions regarding their differential effects. Second, although predefined hierarchies and published data transformations (including rescaling ordinal scale to a continuous 0 to 100 scale) were applied, these assumptions may have influenced effect estimates and introduced additional measurement error. Finally, although analyses were guided by a predefined analysis plan, time point-stratified analyses were introduced post hoc to support interpretation of outcome trajectories. To enhance transparency and methodological rigour, future studies should pre-specify all analyses.

In this review, interpretation of findings was further complicated by the limited reporting of enhanced recovery after surgery (ERAS) protocols, despite their widespread adoption in contemporary surgical care. Heterogeneity in the timing of intervention delivery, with both preoperative and postoperative interventions analysed under a single “(p)rehabilitation” construct, further complicated assessment of outcomes such as postoperative complications and readmissions. In addition, participant adherence, engagement, and digital literacy were not assessed, despite their potential role as important moderators of intervention effectiveness.

### 4.2. Implications for Practice and Research

Technology-enabled (p)rehabilitation appears effective in reducing hospital length of stay and improving some psychosocial outcomes, particularly at later time points. However, benefits across clinical endpoints remain inconclusive. Future research should explore hybrid models that integrate digital and face-to-face delivery, as these approaches may combine the accessibility of technology with the clinical benefits of in-person care. In parallel, studies should investigate which delivery modalities and program characteristics are most effective. Finally, incorporating co-design principles into intervention development may enhance both effectiveness and scalability, as evidence suggests this approach improves adherence, acceptability and patient experience [57,58].

Given the low quality of evidence in this review, appropriately powered RCTs are needed to confirm whether technology-enabled delivery of (p)rehabilitation is effective. To improve evidence quality and reduce heterogeneity across trials, future studies should standardise intervention components. Control groups should consistently follow enhanced recovery after surgery (ERAS) protocols, while intervention groups receive ERAS plus technology-enabled (p)rehabilitation. Trials should also pre-specify primary and secondary outcomes and employ validated outcome measures beyond the immediate perioperative period. Such rigor will enable robust comparisons and strengthen the reliability of future pooled analyses. In addition, future trials should systematically assess and report digital literacy levels of participants, system usability metrics, adherence data (including login frequency and feature utilisation), and technical difficulties encountered. Given the older demographic commonly associated with thoracic and abdominopelvic cancers, understanding these factors is essential for successful implementation and equitable access to digital health interventions. We recommend that standardised reporting frameworks for digital health interventions incorporate these metrics to facilitate meaningful evaluation of intervention feasibility and scalability across diverse populations.

Finally, ensuring equitable access and successful implementation should be integral to future research. Technology-enabled interventions must be accessible across socioeconomic, geographic and cultural contexts to avoid widening disparities. This includes addressing barriers such as language differences and digital literacy. Applying implementation frameworks can support integration into routine practice, promote sustainability and ensure alignment with health system priorities.

## 5. Conclusions

This review enhances the growing evidence base on technology-enabled perioperative care; a rapidly expanding field following the digital transformation of the healthcare setting. The findings suggest that technology-enabled (p)rehabilitation interventions show promise in reducing hospital length of stay and improving selected patient-reported outcomes and experience measures following thoracic and abdominopelvic cancer surgery. However, benefits across selected outcomes are often reported by single studies. Additionally, the quality of evidence is limited by the small number of studies, modest sample sizes, methodological heterogeneity, and variable intervention designs. Large-scale, adequately powered trials are needed to confirm the efficacy of technology-enabled (p)rehabilitation, identify optimal delivery models, and guide future clinical effectiveness and implementation studies.

## Figures and Tables

**Table 1:** Summary of prehabilitation programs included in the review, detailing key study characteristics, intervention components (program type, duration, and delivery mode) and outcomes of interest.

Authors, Year	Characteristics	Intervention Group	Control Group	Outcomes
Huber, 2013 [22]	**Mean age (SD):** 63.3 (7.2) years **Gender, Female**: N/A **Sample Size:** 203 **Type of Cancer:** Prostate Cancer	**Treatment name**: Multimedia-Supported Education (n = 102) **Description**: Standard preoperative education was delivered using a multimedia-supported education tool with an interactive interface that allowed the physician to navigate between illustrations, videos, and textual information. The tool covered topics such as anatomy, the surgical procedure, potential side effects, and general treatment course, including guidance on exercise and nutrition. **Domain:** Physical activity and nutritional support **Provider**: Treating physician **Mode of delivery**: Web-based **Location**: In hospital **Number of sessions**: Single consultation **Duration of session(s)**: 18.8 ± 5.0 min **Intensity**: N/A **Duration of the intervention**: Single preoperative consultation **Tailored**: Yes	**Treatment name**: Standard preoperative education (n = 101) **Description**: Standard preoperative education delivered verbally, with a mean duration of 18.9 ± 5.3 min.	**Anxiety:** State-Trait Anxiety Inventory (STAI) **Patient Satisfaction:** Six-point Likert Scale
Patel, 2023 [23]	**Mean age (SD)**: 67.24 (8.84) years **Gender, Female (%)**: 55 (58%) **Sample size**: 95 **Type of cancer**: Lung Cancer	**Treatment name**: Move For Surgery (n = 45) **Description**: Patients were provided with a wearable activity tracker (Fitbit) alongside printed educational resources. The Fitbit displayed daily reminders to encourage and motivate the participant to reach their daily step goal. **Domain:** Physical activity and nutritional support **Provider**: Research team **Mode of delivery**: Wearable Technology **Location**: At home **Number of sessions**: N/A **Duration of session(s)**: N/A **Intensity**: Step goal was increased by 10% of the baseline each week, capped at 10,000 steps per day. **Duration of the intervention**: 3–4 weeks **Tailored**: Yes	**Treatment name**: Usual preoperative care (n = 50) **Description**: Usual preoperative care, which consisted of education regarding smoking cessation only.	**Length of Hospital Stay** **Health-related Quality of Life:** EuroQol 5-Dimension 5-Level (EQ-5D-5L) Overall Health Component **Pain:** Pain/discomfort score on the EuroQol 5-Dimension 5-Level (EQ-5D-5L)
Rocamora González, 2022 [24]	**Mean age (SD)**: 65.1 years **Gender, Female (%)**: 29 (35.4%) **Sample size**: 82 **Type of cancer**: Colorectal Cancer	**Treatment name**: Calm in the Operating Room App (n = 39) **Description**: The mindfulness-based app included a brief introduction to the Calm Down program and a video familiarising users with the surgical hospital context. It offers two mindfulness training options—the long and short program. The long program, intended for patients with 15 days to one month before surgery, included 14 guided meditation audios. The short program, designed for those with only a few hours or days before surgery, provided 5 guided sessions. All content was developed by professionals accredited in mindfulness teaching. **Domain:** Psychological support **Provider**: Professionals accredited in mindfulness teaching **Mode of delivery**: App-based **Location**: At home **Number of sessions**: Individualised **Duration of session(s)**: Individualised. **Intensity**: N/A **Duration of the intervention**: Up to 1 month **Tailored**: Yes	**Treatment name**: Usual treatment (n = 43) **Description**: Usual treatment, which did not include any protocolised mental health intervention. Patients may have sought psychiatric or psychological treatment independently.	**Health-related Quality of Life:** World Health Organisation Quality of Life- Brief (WHOQOL-BREF) **Anxiety:** Hospital anxiety and depression scale (HADS-A) **Depression:** Hospital anxiety and depression scale (HADS-D) **Patient Satisfaction:** Client Satisfaction Questionnaire (CSQ-8)
Rodriguez, 2023 [25]	**Mean age (SD)**: 62.4 (12.99) years **Gender, Female (%)**: 52 (62.7%) **Sample size**: 83 **Type of cancer**: Pancreatic	**Treatment name**: Telephone-based intervention, in addition to remote monitoring (via a Fitbit) (n = 41) **Description**: The phone intervention was delivered by a specialised physician using a standardised semi-structured script, on average 5.4 ± 1.5 days before surgery. The script began with information on the importance of physical activity, followed by open-ended questions addressing: (1) current activity levels; (2) challenges or barriers to engaging in prehabilitation; (3) available resources and information to improve physical activity; and (4) user functionality of the wearing the device. Based on the patient’s responses, the clinician provided tailored follow-up questions and individualised recommendations. **Domain:** Physical Activity **Provider**: Specialised physician **Mode of delivery**: Telehealth **Location**: At home **Number of sessions**: Single session **Duration of session(s)**: Unspecified **Intensity**: N/A **Duration of the intervention**: Single call **Tailored**: Yes	**Treatment name**: Remote monitoring (via a Fitbit) (n = 42) **Description**: Patients wore a wearable device (Fitbit) to monitor their physical activity. They did not receive any additional intervention.	**Complications:** Modified Accordion Grading System—MAGS
Schmid, 2024 [26]	**Mean age (SD)**:57.0 (13.9) years **Gender, Female (%)**: 67 (100%) **Sample size**: 67 **Type of cancer**: Ovarian, Uterine, Vulva and Cervial Cancer	**Treatment name**: Virtual reality therapy alongside usual care (n = 34) **Description**: The virtual reality (VR) tool presented a 360-degree, 3-dimensional video recording of the real-world environment at the Gold Caost University Hospital, including the pre-operative admission suite, pre-anaesthetic bay, operating theatre, postoperative recovery room and medical staff. **Domain:** Psychological support **Provider**: Unspecified **Mode of delivery**: Virtual reality medium **Location**: In hospital **Number of sessions**: Single exposure session **Duration of session(s)**: 3 min 34 s **Intensity**: N/A **Duration of the intervention**: Single exposure session **Tailored**: No	**Treatment name**: Usual care (n = 33) **Description**: Unspecified	**Anxiety:** Six-tier Visual Facial Anxiety Scale
Shao, 2019 [27]	**Mean age (SD)**: Not reported **Gender, Female (%)**: 33 (25.78%) **Sample size**: 128 **Type of cancer**: Esophageal Squamous Cell Carcinoma	**Treatment name**: Multimedia-based preoperative nursing visit (n = 63) **Description**: An education video on treatment methods for ESCC, benefits of VAST versus open surgery, patient experiences, surgical environment, anaesthesia process, fluid intake, and postoperative care was displayed. This multimedia-based visit occurred the day before surgery. **Domain:** Psychological support **Provider**: Nursing staff **Mode of delivery**: Multimedia video presentation **Location**: In hospital **Number of sessions**: Single Session **Duration of session(s)**: 20-min video **Intensity**: N/A **Duration of the intervention**: Single session; 20-min video **Tailored**: No	**Treatment name**: Usual care (n = 65) **Description**: Usual care delivered one day before surgery	**Anxiety:** Spielberger state-trait anxiety inventory (STAI) and Visual Analog Scale (VAS)
Turrado, 2021 [28]	**Mean age (SD)**: Not reported; median was 65 **Gender, Female (%)**: 46 (36.5%) **Sample size**: 126 **Type of cancer**: Colorectal cancer	**Treatment name**: Virtual Reality Exposure (n = 58) **Description**: An immersive virtual reality (VR) simulation allowed patients to experience each step of the perioperative journey—including the initial surgical consultation, admission into the surgical ward, the operating room, and the postoperative recovery room. This was delivered via a VR software application (VR app) compatible with all major smartphone operating systems and accessed using a VR headset. **Domain:** Psychological support **Provider**: Unspecified **Mode of delivery**: Virtual reality medium **Location**: Unspecified **Number of sessions**: Self-directed and repeatable; patients were granted unrestricted access to the VR app and could engage with the simulation as often as desired. **Duration of session(s)**: 16 min and 34 s (all phases). Specific phases could be selected. **Intensity**: N/A **Duration of the intervention**: Unspecified **Tailored**: No	**Treatment name**: No Virtual Realty exposure (n = 68) **Description**: Standard care was provided.	**Complication:** Overall complication rate **Length of Hospital Stay**
Waller, 2022 [29]	**Mean age (SD)**: 58.25 (10.6) years **Gender, Female (%)**: 11 (50%) **Sample size**: 22 **Type of cancer**: Colorectal adenocarcinoma, Pseudomyxoma Peritonei and Other abdominal cancers	**Treatment name**: Prehabilitation group (n = 11) **Description**: Standard care in addition to a tri-model prehabilitation program delivered through wearable technology (Fitbit) with a digital display and a smartphone application. Participants received an individualised exercise program tailored by a physiotherapist. Nutritional support was provided through written dietary advice and a presentation on pre-operative nutrition. Psychosocial support included daily use of a mindfulness app to provide stress management and relaxation techniques. Standardised structured weekly calls were provided to allow reporting of technical issues and provide tailored prehabilitation support. **Domain:** Physical activity, psychological support, nutritional support **Provider**: Multidisciplinary team **Mode of delivery**: App based **Location**: At home **Number of sessions**: One guided medication daily; weekly calls **Duration of session(s)**: Unspecified **Intensity**: Individualised **Duration of the intervention**: Minimum of two weeks (mean 30.5 days) **Tailored**: Yes	**Treatment name**: Usual care (n = 11) **Description**: Standard care in addition to a wearable device (Fitbit smartwatch) with no display or feedback. The intervention lasted a mean of 20.8 days, with a minimum duration of two weeks.	**Anxiety:** Hospital Anxiety and Depression Scale (HADS-A) **Depression:** Hospital Anxiety and Depression Scale (HADS-D) **Patient Satisfaction:** End of Study Questionnaire

**Table 2:** Summary of rehabilitation programs included in the review, detailing key study characteristics, intervention components (program type, duration, and delivery mode) and outcomes of interest.

Authors, Year	Characteristics	Intervention Group	Control Group	Outcomes
Alves, 2024 [30,31]	**Mean age (SD):** 59.8 (11.3) years **Gender, Female**: 25 (33.3%) **Sample Size:** 70 **Type of Cancer:** Intestinal, Gastric, Other	**Treatment name**: Exergame rehabilitation (n = 35) **Description**: Exergame rehabilitation delivered using four Wii Fit games, in addition to usual care. **Domain:** Physical Activity **Provider**: Healthcare rehabilitation professionals **Mode of delivery**: Videogame **Location**: In hospital **Number of sessions**: 6 **Duration of session(s)**: 15 min on postoperative day 2 and 3, 20 min on postoperative day 4 and 5, and 30 min on postoperative day 6 and 7. **Intensity**: Unspecified **Duration of the intervention**: 6 days **Tailored**: Unspecified	**Treatment name**: Usual care (n = 35) **Description**: Usual care until discharge, including early mobilisation.	**Length of Stay** **Pain:** Numerical Rating Scale **Anxiety:** Hospital Anxiety and Depression Scale (HADS-A) **Depression:** Hospital Anxiety and Depression Scale (HADS-D) **Fatigue:** Fatigue Assessment Scale
Liu, 2024 [32]	**Mean age (SD):** 57.4 (10.4) years **Gender, Female**: 29 (42.6%) **Sample Size:** 68 **Type of Cancer:** Intrahepatic cholangiocarcinoma	**Treatment name**: Online cognitive behavioural stress management (OO-CBSM) program (n = 34) **Description**: Normal care (including telephone follow-ups) in addition to the online cognitive behavioural stress management (OO-CBSM) program. This program included weekly 90-min offline sessions (60 min didactic and 30 min relaxation training) and biweekly online portion via WeChat (including didactic materials, relaxation videos, and doctor–patient communication via sharing session). In addition, patients could communicate with doctors, nurses and other patients in the WeChat group at any time. **Domain:** Psychological support **Provider**: Healthcare team **Mode of delivery**: App-based **Location**: At home **Number of sessions**: Weekly offline sessions and a biweekly online portion **Duration of session(s)**: Weekly 90-min offline sessions (60 min didactic and 30 min relaxation training). Unspecified for the online portion. **Intensity**: Unspecified **Duration of the intervention**: Offline weekly for 10 weeks; online biweekly for 6 months **Tailored**: Yes	**Treatment name**: Normal care and telephone follow-ups (n = 34) **Description**: Normal care (including inpatient postoperative care and the distribution of health education brochures at the time of discharge) in addition to telephone follow-up for six months (weekly for the first 10 weeks, then biweekly thereafter).	**Health-related Quality of Life:** European Organisation for Research and Treatment of Cancer Quality of Life Questionnaire—Core 30 (EORTC QLQ-C30) and EuroQol- 5 Dimension (EQ-5D) Score **Anxiety:** Hospital anxiety and depression scale (HADS-A) and self-rating anxiety scale (SAS) **Depression:** Hospital anxiety and depression scale (HADS-D) and self-rating depression scale (SDS)
Lv, 2024 [33]	**Mean age (SD):** 61 years **Gender, Female**: 77 (56.6%) **Sample Size:** 136 **Type of Cancer:** Lung Cancer	**Treatment name**: Smartphone rehabilitation app (n = 68) **Description**: Usual care plus access to an interactive app. Participants downloaded a smartphone app with three modules: (1) Daily symptom reporting for four core symptoms (pain, coughing, shortness of breath, and fever) which tigered alerts to the medical team if the extent was severe; (2) instructional videos and daily training plans (with reminders) for aerobic and respiratory exercises; (3) educational material covering lung cancer knowledge, surgery perioperative care, importance and methods of rehabilitation, nutritional and psychological support. **Domain:** Physical activity, nutritional support, psychological support **Provider**: Multidisciplinary team, including clinicians and nurses **Mode of delivery**: App-based **Location**: At home **Number of sessions**: Daily exercises and symptom reporting. Educational material could be accessed as needed. **Duration of session(s)**: Unspecified **Intensity**: Tailored **Duration of the intervention**: One month postoperatively **Tailored**: Yes	**Treatment name**: Usual care (n = 68) **Description**: Usual care, including access to an app containing a standard discharge instruction document.	**Complications:** Clavien–Dindo Classification (≥IIIb) **Hospital Readmission:** Hospital readmission within 30 days **Pain:** MD Anderson Symptom Inventory for Lung Cancer (MDASI-LC) **Fatigue:** MD Anderson Symptom Inventory for Lung Cancer (MDASI-LC) **Patient Satisfaction:** Self-designed questionnaire
Schrempf, 2023 [34]	**Mean age (SD)**: 60.65 (9.6) years **Gender, Female (%)**: 25 (40.3%) **Sample size**: 62 **Type of cancer**: Colorectal	**Treatment name**: Immersive Virtual Reality (VR) Group (n = 31) **Description**: Usual care (including physiotherapy) in addition to VR-based bedside fitness exercises using Oculus Quest 2 headset and the Holofit app. Exercise games involved rowing or cycling in virtual environments, requiring active upper body movement. **Domain:** Physical Activity **Provider**: Study staff **Mode of delivery**: Virtual reality medium **Location**: In hospital **Number of sessions**: Once daily on weekdays (Monday to Friday) **Duration of session(s)**: Maximum 30 min per session; supervised first 10 min, then unsupervised **Intensity**: As per American Heart Association Recommendations **Duration of the intervention**: From postoperative day 1 until discharge (mean length of stay 9.0 days in the intervention group) **Tailored**: Yes	**Treatment name**: Usual care (n = 31) **Description**: Usual care, including standard physiotherapy	**Complications:** Clavien–Dindo Classification (≥III) and Comprehensive Complication Index **Length of Hospital Stay** **Health-related Quality of Life:** European Quality of Life 5-Dimension (EQ-5D-5L) Visual Analogue Scale (EQ-VAS) and Index Score **Patient Satisfaction:** European Organisation for Research and Treatment of Cancer (EORTC)
Yu, 2022 [35]	**Mean age (SD)**: Unspecified **Gender, Female (%)**: 29 (17.3%) **Sample size**: 168 **Type of cancer**: Esophageal cancer	**Treatment name**: Telephone and Internet-based supportive care (n = 86) **Description**: Standard care in addition to a nurse led telephone and internet-based supportive care (via a WeChat group). Prior to discharge, patients joined a nurse-led WeChat group where they could ask questions at any time. Nurses responded daily and called patients regularly. After discharge, nurses conducted structured one-on-one phone calls: weekly (months 1–2), biweekly (months 3–4), and monthly (months 5–6). These 20–30 min calls addressed nutrition (using the SDSAT tool), postoperative symptoms (e.g., pain, reflux), and psychological support. Nurses answered patient questions and provided tailored advice and counselling as needed. The WeChat group also enabled patients to share images and videos, connect with peers, and access resources. Nurses shared FAQs, information on oral nutritional supplements, and articles on rehabilitation and nutrition. **Domain:** Nutritional and psychological support **Provider**: Supportive care team **Mode of delivery**: App based **Location**: At home **Number of sessions**: 14 (telephone calls) **Duration of session(s)**: 20–30 min (telephone calls) **Intensity**: N/A **Duration of the intervention**: 6 months **Tailored**: Yes	**Treatment name**: Conventional care (n = 82) **Description**: Standard care (including outpatient clinic visits 1 month after discharge and then once every 3 months for 2 years, once every 6 months in year 2–5 and once a year after 5 years) in addition to telephone follow-up with the physician assistance once every 3 months to confirm the patient’s situation and answer questions. Patients could also contact the physician assist as required.	**Health-related Quality of Life:** European Organisation for Research and Treatment of Cancer Quality of Life Questionnaire—Core 30 (EORTC QLQ-C30) **Pain:** Symptom scale of the European Organisation for Research and Treatment of Cancer Quality of Life Questionnaire—Core 30 (EORTC QLQ-C30) **Fatigue:** Symptom scale of the European Organisation for Research and Treatment of Cancer Quality of Life Questionnaire—Core 30 (EORTC QLQ-C30) **Patient Satisfaction:** Likert Scales

**Table 3:** Summary of combined prehabilitation and rehabilitation programs included in the review, detailing key study characteristics, intervention components (program type, duration, and delivery mode) and outcomes of interest.

Authors, Year	Characteristics	Intervention Group	Control Group	Outcomes
Low, 2023 [36]	**Mean age (SD):** 56.2 (10.5) years **Gender, Female**: 11 (42.3%) **Sample Size:** 26 **Type of Cancer:** Metastatic gastrointestinal and peritoneal cancer	**Treatment name**: Detecting Activity to Support Healing (DASH) intervention (n = 13) **Description**: Activity monitoring plus the sedentary behaviour (SB) intervention. The SB intervention utilised a Fitbit smartwatch and a smartphone app (DASH) that sent activity prompts when prolonged sedentary behaviour was detected. Prompt frequency was tailored based on daily symptom severity (which was self-reported by patients’ on the app) and between each participant’s walking time and bedtime. **Domain:** Physical activity **Provider**: Unspecified **Mode of delivery**: App-based **Location**: During inpatient stay (as feasible) and at home (preoperatively and post-discharge) **Number of sessions**: N/A **Duration of session(s)**: N/A **Intensity**: Individualised **Duration of the intervention**: 44–92 days (an average of 57.2 days was reported) **Tailored**: Yes	**Treatment name**: Activity monitoring only (n = 13) **Description**: Participants received a Fitbit smartwatch app that measured steps. No activity prompts were sent.	**Hospital Readmission:** Hospital readmission within 30 days post index hospital discharge **Health-related Quality of Life:** Functional Assessment of Cancer Therapy (FACT) **Depression:** Center for Epidemiological Studies-Depression (CES-D) **Patient satisfaction:** End-of-study interview
Min, 2024 [37]	**Mean age (SD):** 61.3 (8.1) years **Gender, Female**: 36 (30.5%) **Sample Size:** 118 **Type of Cancer:** Esophageal Cancer	**Treatment name**: Internet and rehabilitation guidance (n = 59) **Description**: Usual care in addition to a rehabilitation guidance intervention delivered via the WeChat platform. The intervention was based on the IKAP framework. Preoperatively, knowledge was imparted via the hospital’s WeChat public account which contained health education articles (1–3 additional articles were published weekly). Additionally, clinician-led health education lectures were delivered twice weekly (Monday & Wednesday), covering surgical procedures, risks, and perioperative care. Photos of the operating room were also shared to familiarise patients with the surgical environment. Postoperatively, regular WeChat messages reinforced postoperative precautions and provided guidance and reminders related to graded physical activity, sleep hygiene, and dietary adjustment. Additionally, a WeChat support group was created to facilitate peer interaction, with recovered patients sharing positive experiences to foster optimism. At home, patients were contacted weekly on WeChat to monitor symptoms and habits. Ongoing health education articles (on self-care skills and complications) were delivered via the WeChat public account and WeChat one-on-one messaging. Individual behavioural coaching was delivered via one-on-one voice messages to address lifestyle modification for those with poor habits (e.g., irregular sleep or diet). **Domain:** Physical Activity, psychological support, nutritional support **Provider**: Multidisciplinary team **Mode of delivery**: App-based **Location**: In hospital and at home **Number of sessions**: N/A **Duration of session(s)**: N/A **Intensity**: N/A **Duration of the intervention**: Unspecified **Tailored**: Yes	**Treatment name**: Routine care (n = 59) **Description**: Routine perioperative nursing, including preoperative operation and disease-related health education, postoperative precautions and dietary guidance before discharge.	**Complications:** Total Postoperative Complications (30 days post index surgery) **Length of Hospital stay**
Schrempf, 2022 [38]	**Mean age (SD)**: 58.4 (10.35) years **Gender, Female (%)**: 17 (47.2%) **Sample size**: 36 **Type of cancer**: Colorectal and liver metastases (from colorectal cancer)	**Treatment name**: Virtual Reality Group (n = 18) **Description**: Patients engaged in immersive, mindfulness-based virtual reality sessions designed to promote relaxation. These sessions incorporated guided reflections, breathing exercises with visual feedback, binaural audio, meditative music, and interactive mini-games. The morning session focused on attentional engagement, while the evening session emphasised calming guided meditations. **Domain:** Psychological support **Provider**: Study staff **Mode of delivery**: Virtual Reality medium **Location**: In hospital **Number of sessions**: Twice daily (morning and evening) from Monday to Friday, beginning preoperatively (on the day of admission or the day of surgery for patients undergoing afternoon surgery) and continued postoperatively until hospital discharge. **Duration of session(s)**: Each session lasted approximately 7–10 min (morning session 7–8 min, evening session 10 min) **Intensity**: N/A **Duration of the intervention**: Preoperatively (on the day of admission or the day of surgery for patients undergoing afternoon surgery) until hospital discharge. **Tailored**: No	**Treatment name**: Standard Care (n = 18) **Description:** Standard care (no intervention).	**Complications:** Clavien–Dindo Classification and Comprehensive Complication Index (CCI) **Length of Hospital Stay** **Patient Satisfaction:** Study specific questionnaire
Yuan, 2023 [39]	**Mean age (SD)**: Not reported **Gender, Female (%)**: 129 (64.5%) **Sample size**: 200 **Type of cancer**: Lung Cancer	**Treatment name**: Multimodal health education combined with feedback (n = 100) **Description**: Patients participated in a multimodal perioperative health education program based on the Clinical Practice Guidelines for ERAS in China (2021 edition) and the common clinical issues observed in lung cancer patients. Upon admission, each patient received a copy of the Manual of Rapid Rehabilitation of Thoracic Surgery, which was compiled in accordance with these guidelines and issues. The manual covered key topics across admission, preoperative, postoperative, and discharge education, with a focus on safety, exercise, nutrition, and recovery. A complementary perioperative health education video reinforced these themes, addressing admission procedures, preoperative preparation, postoperative precautions, functional exercises, airway clearing and discharge guidance. The video was easily accessible via a QR code displayed in wards and shared through the hospital’s WeChat patient group. During hospitalisation, nurses delivered education through a combination of group sessions (using PowerPoint and video presentations) and individualised one-on-one education. **Domain:** Physical Activity and nutritional support **Provider**: Multidisciplinary team **Mode of delivery**: App-based **Location**: In hospital **Number of sessions**: Unspecified **Duration of session(s)**: Unspecified **Intensity**: N/A **Duration of the intervention**: Unspecified **Tailored**: Yes	**Treatment name**: Routine Health Education (n = 100) **Description**: Routine health education including smoking cessation advice, preoperative respiratory exercises and perioperative precautions.	**Length of Hospital Stay** **Distress:** Huaxi Emotional-Distress Index **Patient Satisfaction:** Nursing Satisfaction Score

**Table 4:** Summary of findings and quality of evidence assessment (GRADE).

Timepoint [Author, Year]	Summary of Findings		Quality of Evidence Assessment (GRADE)				
	Sample (Studies)	Effect Size (95%CI)	Risk of Bias	Inconsistency	Imprecision	Publication Bias	Overall Quality of Evidence
**Postoperative Complications**							
[Lv, 2024 [33]; Min, 2024 [37]; Rodriguez, 2023 [25]; Schrempf, 2022 [38]; Schrempf, 2023 [34]; Turrado, 2020 [28]]	552 (6 RCTs)	RR: 0.95 (0.69 to 1.32)	Serious	Serious	Not serious	Undetected	Low
**Hospital Readmission**							
**Within 30 Days** [Low, 2023 [36]; Lv, 2024 [33]]	162 (2 RCTs)	RR: 1.46 (0.57 to 3.76)	Serious	Not serious	Serious	Undetected	Low
**Length of Hospital Stay**							
[Alves, 2024 [30,31]; Min 2024 [37]; Patel, 2023 [23]; Schrempf, 2022 [38]; Schrempf, 2023 [34]; Turrado, 2020 [28]; Yuan, 2023 [39]]	707 (7 RCTs)	MD: 1.33 (0.59 to 2.07)	Serious	Not serious	Not serious	Undetected	Moderate
**Quality of Life**							
**Baseline** [Liu, 2024 [32]; Patel, 2023 [23]; Rocamora González, 2022 [24]; Schrempf, 2023 [34]]	307 (4 RCTs)	MD: −0.58 (−1.68 to 0.51)	Serious	Not serious	Serious	Undetected	Low
**Preoperatively** [Low, 2023 [36]]	26 (1 RCT)	MD: 2.78 (−3.89 to 9.45)	Not serious	Not serious	Serious	Undetected	Low
**Immediate Postoperative Period** [Low, 2023 [36]; Patel, 2023 [23]; Rocamora González, 2022 [24]; Schrempf, 2023 [34]]	265 (4 RCTs)	MD: 1.33 (−3.49 to 6.15)	Serious	Serious	Serious	Undetected	Very Low
**Early Postoperative Period** [Liu, 2024 [32]; Low, 2023 [36]; Rocamora González, 2022 [24]; Schrempf, 2023 [34]]	238 (4 RCTs)	MD: −0.25 (−1.39 to 0.89)	Not serious	Serious	Serious	Undetected	Low
**Intermediate Postoperative Period** [Liu, 2024 [32]]	68 (1 RCT)	MD: 7.50 (0.65 to 14.35)	Not serious	Not serious	Serious	Undetected	Low
**Long-term Postoperative Period** [Liu, 2024 [32]; Yu, 2022 [35]]	236 (2 RCTs)	MD: 9.93 (4.34 to 15.51)	Not serious	Not serious	Serious	Undetected	Moderate
**Pain**							
**Baseline** [Alves, 2024 [30]; Lv, 2024 [33]]	206 (2 RCTs)	MD: −1.77 (−7.74 to 4.19)	Serious	Serious	Serious	Undetected	Very Low
**Immediate Postoperative Period** [Alves, 2024 [30]; Lv, 2024 [33]; Patel, 2023 [23]]	301 (3 RCTs)	MD: 12.18 (7.19 to 17.17)	Serious	Not serious	Serious	Undetected	Low
**Early Postoperative Period** [Lv, 2024 [33]]	136 (1 RCT)	MD: 3.40 (−2.11 to 8.91)	Serious	Not serious	Serious	Undetected	Very Low
**Long-term Postoperative Period** [Yu, 2022 [35]]	168 (1 RCT)	MD: 8.10 (2.71 to 13.49)	Not serious	Not serious	Serious	Undetected	Low
**Anxiety**							
**Baseline** [Alves, 2024 [30]; Liu, 2024 [32]; Rocamora González, 2022 [24]; Shao, 2019 [27]; Schmid, 2024 [26]; Waller, 2022 [29]]	437 (6 RCTs)	MD: −2.10 (−5.53 to 1.34)	Not serious	Serious	Not serious	Undetected	Moderate
**Preoperatively** [Huber, 2013 [22]; Shao, 2019 [27]; Schmid, 2024 [26]; Waller, 2022 [29]]	420 (4 RCTs)	MD: 11.83 (−0.18 to 23.84)	Not serious	Not serious	Not serious	Undetected	High
**Immediate Postoperative Period** [Alves, 2024 [30]; Rocamora González, 2022 [24]; Shao, 2019 [27]]	280 (3 RCTs)	MD: 2.30 (−8.97 to 13.57)	Serious	Serious	Serious	Undetected	Very Low
**Early Postoperative Period** [Liu, 2024 [32]; Rocamora González, 2022 [24]]	150 (2 RCTs)	MD: −0.90 (−9.25 to 7.44)	Not serious	Serious	Serious	Undetected	Low
**Intermediate Postoperative Period** [Liu, 2024 [32]]	68 (1 RCT)	MD: 5.20 (0.22 to 10.18)	Not serious	Not serious	Serious	Undetected	Low
**Long-term Postoperative Period** [Liu, 2024 [32]]	68 (1 RCT)	MD: 7.62 (2.64 to 12.60)	Not serious	Not serious	Serious	Undetected	Low
**Depression**							
**Baseline** [Alves, 2024 [30]; Liu, 2024 [32]; Rocamora González, 2022 [24]; Waller, 2022 [29]]	242 (4 RCTs)	MD: −1.83 (−5.83 to 3.07)	Serious	Serious	Serious	Undetected	Very Low
**Preoperatively** [Waller, 2022 [29]; Low, 2023 [36]]	48 (2 RCT)	MD: 3.35 (−1.62 to 8.33)	Not serious	Not serious	Serious	Undetected	Moderate
**Immediate Postoperative Period** [Alves, 2024 [30]; Rocamora González, 2022 [24]; Low, 2023 [36]]	178 (3 RCTs)	MD: 2.09 (−3.06 to 7.24)	Serious	Serious	Serious	Undetected	Very Low
**Early Postoperative Period** [Liu, 2024 [32]; Low, 2023 [36]; Rocamora González, 2022 [24]]	176 (3 RCTs)	MD: 3.27 (−2.85 to 9.39)	Not serious	Serious	Serious	Undetected	Low
**Intermediate Postoperative Period** [Liu, 2024 [32]]	68 (1 RCT)	MD: 6.67 (1.01 to 12.33)	Not serious	Not serious	Serious	Undetected	Low
**Long-term Postoperative Period** [Liu, 2024 [32]]	68 (1 RCT)	MD: 6.19 (−0.04 to 12.42)	Not serious	Not serious	Serious	Undetected	Low
**Fatigue**							
**Baseline** [Alves, 2024 [30]; Lv, 2024 [33]]	206 (2 RCTs)	MD: 3.79 (−6.68 to 14.26)	Serious	Serious	Serious	Undetected	Very Low
**Immediate Postoperative Period** [Alves, 2024 [30]; Lv, 2024 [33]]	206 (2 RCTs)	MD: 4.28 (−9.35 to 17.91)	Serious	Serious	Serious	Undetected	Very Low
**Early Postoperative Period** [Lv, 2024 [33]]	136 (1 RCT)	MD: 3.80 (−0.96 to 8.56)	Serious	Not serious	Serious	Undetected	Very Low
**Long-term Postoperative Period** [Yu, 2022 [35]]	168 (1 RCT)	MD: 18.57 (13.77 to 23.37)	Not serious	Not serious	Serious	Undetected	Low
**Distress**							
**Early Postoperative Period** [Yuan, 2023 [39]]	200 (1 RCT)	MD: 1.23 (0.30 to 2.16)	Not serious	Not serious	Serious	Undetected	Low

## Data Availability

All data for this meta-analysis are publicly available within the included publications.

## Supplementary Materials

The following supporting information can be downloaded at: https://www.mdpi.com/article/10.3390/cancers18020296/s1, Table S1: PRISMA 2020 Checklist.; Table S2: Search Strategy conducted in MEDLINE/EMBASE (via Ovid).; Table S3: Transformation and standardisation of reported outcomes across included studies, stratified by program type. Ordinal scales were standardised to continuous 0–100 scales to facilitate comparability across instruments. Outcome measures for hospital readmission, distress and patient satisfaction were not reported as no transformation or standardisation was required.; Table S4: Excluded outcome measures, including reason for exclusion, stratified by program type.; Table S5A: Definitions of assessment timepoints used in the included studies.; Table S5B: Reported assessment timepoint in included studied, stratified by program type. Reported timepoints for patient satisfaction are excluded.; Table S6A: Hierarchical prioritisation of outcome measures used in the included studies.; Table S6B: Hierarchical prioritisation of assessment timepoints used in the included studies.; Table S7: Excluded publications, including reasoning.; Figure S1: Risk of postoperative complications and hospital readmission. Risk ratios <1 favours prehabilitation interventions.; Figure S2: Mean difference for postoperative length of hospital stay (days) in randomised controlled trials of technology-enabled (p)rehabilitation for patients undergoing thoracic and/or abdominopelvic cancer surgery. Positive values favour prehabilitation interventions.; Figure S3: Mean difference in health-related quality of life in randomised controlled trials of technology-enabled (p)rehabilitation for patients undergoing thoracic and/or abdominopelvic cancer surgery. Positive values favour prehabilitation interventions.; Figure S4: Mean difference in pain in randomised controlled trials of technology-enabled (p)rehabilitation for patients undergoing thoracic and/or abdominopelvic cancer surgery. Positive values favour prehabilitation interventions.; Figure S5: Mean difference in anxiety in randomised controlled trials of technology-enabled (p)rehabilitation for patients undergoing thoracic and/or abdominopelvic cancer surgery. Positive values favour prehabilitation interventions.; Figure S6: Mean difference in fatigue in randomised controlled trials of technology-enabled (p)rehabilitation for patients undergoing thoracic and/or abdominopelvic cancer surgery. Positive values favour prehabilitation interventions.; Figure S7: Mean difference in distress in randomised controlled trials of technology-enabled (p)rehabilitation for patients undergoing thoracic and/or abdominopelvic cancer surgery. Positive values favour prehabilitation interventions.; Table S8: Patient satisfaction outcomes reported in the included studies, stratified by program type.

## Abbreviations

The following abbreviations are used in this manuscript:

RCTs	Randomised Controlled Trials
LOS	Length of Stay
QoL	Quality of Life
RR	Relative Risks
MD	Mean Differences
CI	Confidence Intervals
PROMs	Patient Reported Outcome Measures
PREMs	Patient Reported Experience Measures
RoB 2	Version 2 of the Cochrane Risk-of-Bias Tool for Randomised Trials
GRADE	Grading of Recommendations, Assessment, Development, and Evaluations
ERAS	Enhanced Recovery After Surgery
