# Supplementary material for: Technology-Enabled (P)rehabilitation for Patients Undergoing Cancer Surgery: A Systematic Review and Meta-Analysis
Source: Cancers (Basel). 2026 Jan 18;18(2):296. doi: 10.3390/cancers18020296 (PMC12839342; doi:10.3390/cancers18020296)
Supplement: Supplementary file 1 [file cancers-18-00296-s001.zip › cancers-4076023-supplementary.pdf]

Supplementary Information

Table of Contents

Table S1 – PRISMA 2020 Checklist 2

Table S2 – Search Strategy 5

Table S3 – Transformation and Standardisation of Reported Outcomes 6

Table S4 – Excluded Outcome Measures 9

Table S5– Timepoints 10

Table S5A – Definitions 10

Table S5B – Timepoints reported in the included studies 11

Table S6 – Prioritisation of timepoints: 13

Material S1 – Prioritisation of outcomes for analysis 13

Material S1 – Prioritisation of measurements: 13

Supplementary Figures 14

Figure S1 – Postoperative Complication and Hospital Readmission 14

Figure S2 – Hospital Length of Stay 15

Figure S3 – Health-Related Quality of Life 16

Figure S4 – Pain 17

Figure S5 – Anxiety 18

Figure S6 – Fatigue 19

Figure S7 – Distress 19

Table S1 – PRISMA 2020 Checklist

Table S1: PRISMA 2020 Checklist

Section and Topic	Item #	Checklist item	Location where item is reported
**TITLE**			
Title	1	Identify the report as a systematic review.	Page 1
**ABSTRACT**			
Abstract	2	See the PRISMA 2020 for Abstracts checklist.	Pages 2-3
**INTRODUCTION**			
Rationale	3	Describe the rationale for the review in the context of existing knowledge.	Page 4
Objectives	4	Provide an explicit statement of the objective(s) or question(s) the review addresses.	Page 4
**METHODS**			
Eligibility criteria	5	Specify the inclusion and exclusion criteria for the review and how studies were grouped for the syntheses.	Pages 5-6
Information sources	6	Specify all databases, registers, websites, organisations, reference lists and other sources searched or consulted to identify studies. Specify the date when each source was last searched or consulted.	Page 4
Search strategy	7	Present the full search strategies for all databases, registers and websites, including any filters and limits used.	Supplementary Appendix 2
Selection process	8	Specify the methods used to decide whether a study met the inclusion criteria of the review, including how many reviewers screened each record and each report retrieved, whether they worked independently, and if applicable, details of automation tools used in the process.	Page 5
Data collection process	9	Specify the methods used to collect data from reports, including how many reviewers collected data from each report, whether they worked independently, any processes for obtaining or confirming data from study investigators, and if applicable, details of automation tools used in the process.	Page 5
Data items	10a	List and define all outcomes for which data were sought. Specify whether all results that were compatible with each outcome domain in each study were sought (e.g. for all measures, time points, analyses), and if not, the methods used to decide which results to collect.	Pages 5-6 & Supplementary Appendix 4-6
	10b	List and define all other variables for which data were sought (e.g. participant and intervention characteristics, funding sources). Describe any assumptions made about any missing or unclear information.	Pages 5-6
Study risk of bias assessment	11	Specify the methods used to assess risk of bias in the included studies, including details of the tool(s) used, how many reviewers assessed each study and whether they worked independently, and if applicable, details of automation tools used in the process.	Page 6
Effect measures	12	Specify for each outcome the effect measure(s) (e.g. risk ratio, mean difference) used in the synthesis or presentation of results.	Pages 6-7

Section and Topic	Item #	Checklist item	Location where item is reported
Synthesis methods	13a	Describe the processes used to decide which studies were eligible for each synthesis (e.g. tabulating the study intervention characteristics and comparing against the planned groups for each synthesis (item #5)).	Pages 5-6 & Supplementary Appendix 4
	13b	Describe any methods required to prepare the data for presentation or synthesis, such as handling of missing summary statistics, or data conversions.	Pages 5-6 & Supplementary Appendix 3
	13c	Describe any methods used to tabulate or visually display results of individual studies and syntheses.	Pages 5-6
	13d	Describe any methods used to synthesize results and provide a rationale for the choice(s). If meta-analysis was performed, describe the model(s), method(s) to identify the presence and extent of statistical heterogeneity, and software package(s) used.	Pages 6-7
	13e	Describe any methods used to explore possible causes of heterogeneity among study results (e.g. subgroup analysis, meta-regression).	Page 6
	13f	Describe any sensitivity analyses conducted to assess robustness of the synthesized results.	N/A
Reporting bias assessment	14	Describe any methods used to assess risk of bias due to missing results in a synthesis (arising from reporting biases).	N/A
Certainty assessment	15	Describe any methods used to assess certainty (or confidence) in the body of evidence for an outcome.	Page 6
**RESULTS**			
Study selection	16a	Describe the results of the search and selection process, from the number of records identified in the search to the number of studies included in the review, ideally using a flow diagram.	Page 7
	16b	Cite studies that might appear to meet the inclusion criteria, but which were excluded, and explain why they were excluded.	Supplementary Appendix 4
Study characteristics	17	Cite each included study and present its characteristics.	Pages 7-23
Risk of bias in studies	18	Present assessments of risk of bias for each included study.	Page 24
Results of individual studies	19	For all outcomes, present, for each study: (a) summary statistics for each group (where appropriate) and (b) an effect estimate and its precision (e.g. confidence/credible interval), ideally using structured tables or plots.	Pages 29-33 & Supplementary Appendix 7
Results of syntheses	20a	For each synthesis, briefly summarise the characteristics and risk of bias among contributing studies.	Page 24
	20b	Present results of all statistical syntheses conducted. If meta-analysis was done, present for each the summary estimate and its precision (e.g. confidence/credible interval) and measures of statistical heterogeneity. If	Pages 29-33 & Supplementary

Section and Topic	Item #	Checklist item	Location where item is reported
		comparing groups, describe the direction of the effect.	Appendix 7
	20c	Present results of all investigations of possible causes of heterogeneity among study results.	Pages 7-8
	20d	Present results of all sensitivity analyses conducted to assess the robustness of the synthesized results.	N/A
Reporting biases	21	Present assessments of risk of bias due to missing results (arising from reporting biases) for each synthesis assessed.	N/A
Certainty of evidence	22	Present assessments of certainty (or confidence) in the body of evidence for each outcome assessed.	Pages 26-28
**DISCUSSION**			
Discussion	23a	Provide a general interpretation of the results in the context of other evidence.	Pages 33-35
	23b	Discuss any limitations of the evidence included in the review.	Pages 34-35
	23c	Discuss any limitations of the review processes used.	Pages 34-35
	23d	Discuss implications of the results for practice, policy, and future research.	Pages 35-36
**OTHER INFORMATION**			
Registration and protocol	24a	Provide registration information for the review, including register name and registration number, or state that the review was not registered.	Page 4
	24b	Indicate where the review protocol can be accessed, or state that a protocol was not prepared.	Page 4
	24c	Describe and explain any amendments to information provided at registration or in the protocol.	Page 35
Support	25	Describe sources of financial or non-financial support for the review, and the role of the funders or sponsors in the review.	Page 38
Competing interests	26	Declare any competing interests of review authors.	Page 38
Availability of data, code and other materials	27	Report which of the following are publicly available and where they can be found: template data collection forms; data extracted from included studies; data used for all analyses; analytic code; any other materials used in the review.	Page 38

Table S2 – Search Strategy

Table S2: Search Strategy conducted in MEDLINE / EMBASE (via Ovid)

MEDLINE/EMBASE via Ovid	
#1	(exp preoperative exercise/ or preoperative exercise.mp. or preoperative exercise/ or exercis*.mp. or exercise/ or physical fitness.mp. or physical fitness/ or exercis* therap*.mp. or exercise therapy/ or physical activity.mp. or exp rehabilitation/ or rehab*.mp. or rehabilitation/ or exercise training.mp. or physical therap*.mp. or physiotherapy.mp.)
#2	(exp diet/ or diet*.mp. or nutrition*.mp. or diet* counsel*.mp. or exp enteric feeding/ or enter* nutrition*.mp. or exp diet therapy/ or nutrition* therap*.mp. or exp parenteral nutrition/ or exp nutritional support/ or nutrition* support.mp. or exp food/)
#3	(exp cognitive behavioral therapy/ or cognitive behavio*ral therap*.mp. or relaxation.mp. or exp mindfulness/ or mindfulness.mp. or exp coping behavio*r/ or coping.mp. or psycho* intervention*.mp. or exp psychotherapy/ or psychotherap*.mp. or CBT.tw. or exp mental health/ or psycho*.mp. or psychoeducation/)
#4	(exp randomized controlled trial/ or randomi*ed controlled trial.mp. or exp controlled clinical trial/ or controlled clinical trial.mp. or randomi*ed.tw. or clinical trial.tw. or trial.tw. or quasi-randomi*ed.tw. or placebo.tw. or random*.tw. or RCT.tw.)
#5	(exp neoplasm/ or neoplas*.mp. or neoplasm/ or malignan*.mp. or malignancy/ or cancer*.mp. or tumo*r.mp.)
#6	(exp abdominal surgery/ or abdo* surg*.mp. or abdominal surgery/ or exp pelvic surgery/ or pelvic surg*.mp. or pelvic surgery/ or exp thorax surgery/ or thora* surg*.mp. or thoracic surgery/)
#7	(exp telemedicine/ or telemedicine.mp. or telemedicine/ or exp mobile phone/ or mobile phone.mp. or mobile phone/ or exp mobile application/ or mobile app*.mp. or smartphone/ or exp technology/ or technology.mp. or technology/ or exp medical technology/ or medical technolog*.mp. or medical technology/ or exp mobile application/ or mobile app*.mp. or exp mobile phone/ or mobile phone.mp. or exp smart phone/ or smartphone.mp. or smartphone/ or exp internet/ or internet.mp. or internet/ or exp computer/ or computer.mp. or computer/ or mHealth.mp. or exp video game/ or video gam*.mp. or video game/ or exp virtual reality/ or virtual reality.mp. or virtual reality/)
#8	#1 OR #2 OR #3
#9	#4 AND #5 AND #6 AND #7 AND #8

Table S3 – Transformation and Standardisation of Reported Outcomes

Table S3: Transformation and standardisation of reported outcomes across included studies, stratified by program type. Ordinal scales were standardised to continuous 0-100 scales to facilitate comparability across instruments. Outcome measures for hospital readmission, distress and patient satisfaction were not reported as no transformation or standardisation was required.

Authors, Year	Outcomes					
	Hospital Length of Stay	Quality of Life	Pain	Anxiety	Depression	Fatigue
**Prehabilitation Programs**						
**Huber, 2013**				**Standardisation:** Scores were rescaled to a 0 – 100 range		
**Patel, 2023**	No transformations or standardisations were applied	No transformation or standardisation were applied	**Transformation:** Medians and interquartile ranges were converted to means and standard deviations. **Standardisation:** Scores were rescaled to a 0–100 range.			
**Rocamora González, 2022**		No transformations or standardisations were applied		**Standardisation:** Scores were rescaled to a 0 – 100 range	**Standardisation:** Scores were rescaled to a 0 – 100 range	
**Rodriguez, 2023**						
**Schmid, 2024**				**Transformation:** Medians and interquartile ranges were converted to means and standard deviations. **Standardisation:** Scores were rescaled to a 0–100 range.		
**Shao, 2019**				**Standardisation:** Scores were rescaled to a 0 – 100 range		
**Turrado, 2021**	**Transformation:** Medians and 95% confidence intervals were converted to means and standard deviations.					

**Waller, 2022**				**Transformation:** 95% confidence intervals were converted to standard deviations. **Standardisation:** Scores were rescaled to a 0–100 range.	**Transformation:** 95% confidence intervals were converted to standard deviations. **Standardisation:** Scores were rescaled to a 0–100 range.	
**Rehabilitation Programs**						
**Alves, 2024**	No transformations or standardisations were applied		**Standardisation:** Scores were rescaled to a 0 – 100 range	**Standardisation:** Scores were rescaled to a 0 – 100 range	**Standardisation:** Scores were rescaled to a 0 – 100 range	**Standardisation:** Scores were rescaled to a 0 – 100 range
**Liu, 2024**		**Standardisation:** Scores were rescaled to a 0 – 100 range		**Standardisation:** Scores were rescaled to a 0 – 100 range	**Standardisation:** Scores were rescaled to a 0 – 100 range	
**Lv, 2024**			**Standardisation:** Scores were rescaled to a 0 – 100 range			**Standardisation:** Scores were rescaled to a 0 – 100 range
**Schrempf, 2023**	No transformations or standardisations were applied	**Standardisation:** Scores were rescaled to a 0 – 100 range				
**Yu, 2022**		**Transformation:** Summary scores were calculated according to the manual, and standard deviations were calculated using a pooled approached	No transformations or standardisations were applied			No transformations or standardisations were applied
**Both Prehabilitation and Rehabilitation**						
**Low, 2023**		**Standardisation:** Scores were rescaled to a 0 – 100 range			**Standardisation:** Scores were rescaled to a 0 – 100 range	
**Min, 2024**	No transformations or standardisations were applied					
**Schrempf, 2022**	**Transformation:** Medians and interquartile ranges were converted to means and standard deviations.					

**Yuan, 2023**	No transformations or standardisations were applied					

Table S4 – Excluded Outcome Measures

Table S4: Excluded outcome measures, including reason for exclusion, stratified by program type.

Authors, Year	Outcome(s)	Reason for exclusion
**Prehabilitation Programs**		
**Rocamora González, 2022**	**Pain:** VAS	No effect size reported
**Turrado, 2021**	**Anxiety:** Hospital anxiety and depression scale (HADS-A)<br><br>**Depression:** Hospital anxiety and depression scale (HADS-D)	Data was reported in an unclear format
**Rehabilitation Programs**		
**Chang, 2020** *	**Complications:** Clavien-Dindo Classification	Outcome was measured prior to intervention initiation
	**Health-related Quality of Life:** European Organisation for Research and Treatment of Cancer Quality of Life Questionnaire – Core 30 (EORTC QLQ-C30)<br><br>**Pain:** Symptom scale of EORTC QLQ-C30<br><br>**Fatigue:** Symptom scale of EORTC QLQ-C30	Data was reported following analysis using generalised estimating equations (GEE)
**Jiang, 2023** **	**Health-related Quality of Life:** European Organisation for Research and Treatment of Cancer Quality of Life Questionnaire – Core 30 (EORTC QLQ-C30)	Data was reported following analysis using generalised estimating equations (GEE) with Bonferroni corrections
**Lv, 2024**	**Length of Stay**	Outcome was measured prior to intervention initiation
**Schrempf, 2023**	**Distress:** National Comprehensive Cancer Network (NCCN) Distress Thermometer	No effect size reported
**Both Prehabilitation and Rehabilitation**		
**Schrempf, 2022**	**Health-related Quality of Life:** European Organisation for Research and Treatment of Cancer Quality of Life Questionnaire – Core 30 (EORTC QLQ-C30)<br><br>**Pain:** Symptom scale of EORTC QLQ-C30<br><br>**Fatigue:** Symptom scale of EORTC QLQ-C30	Data was reported in a format unsuitable for extraction

**This publication was excluded as all relevant outcomes were not suitable for data extraction. The study evaluated a 12-week exercise and nursing education health informatics program delivered via an app in patients with oesophageal cancer (n = 88; mean age 56.0 ± 9.4 years; 9.1% female). Participants in the intervention group (n = 44) received usual care plus a moderate-intensity walking regimen (30 minutes per session, 3–4 sessions per week) monitored using a smart bracelet, alongside nurse-led education delivered through e-books. The health informatics component involved an application that enabled patients to communicate questions or concerns to nurse practitioners, receive immediate guidance, and participate in an online discussion group. The control group (n = 44) received usual postoperative care, including conventional postoperative feeding, wound care, and daily rehabilitation exercises.*

***This publication was excluded as all relevant outcomes were not reported in a form suitable for data extraction. The study evaluated the iNutrition intervention, an individualised mHealth nutrition program delivered via a WeChat app ("iNutrition applet") and biweekly telephone consultations (mean duration 23.60 ± 8.94 minutes) over 12 weeks in patients with gastric cancer (n = 24; mean age*

54.9 ± 10.3 years; 33.3% female). The control group received usual care, including printed nutrition education materials and postoperative nutrition advice.

Table S5– Timepoints

Table S5A – Definitions

Table S5A: Definitions of assessment timepoints used in the included studies.

Timepoint Label	Timepoints definition
**Baseline**	Before intervention delivery
**Preoperative**	Anytime before surgery
**Immediate Postoperative Period**	Post surgery to 7 days postoperatively
**Early Postoperative Period**	>7 days to 1-month post-surgery
**Intermediate Postoperative Period**	>1 month to 3 months post-surgery
**Long-Term Postoperative Period**	>3 months post-surgery

Table S5B – Timepoints reported in the included studies

Table S5B: Reported assessment timepoint in included studies, stratified by program type. Reported timepoints for patient satisfaction are excluded.

Authors, Year	Timepoints					
	Baseline	Preoperative	Immediate Postoperative Period	Early Postoperative Period	Intermediate Postoperative Period	Long-Term Postoperative Period
**Prehabilitation Programs**						
**Huber, 2013**		Within 6-10 hrs after intervention delivery				
**Patel, 2023**	Baseline		Postoperative Day 1			
**Rocamora González, 2022**	Baseline (T0)		Discharge from Hospital (T1)	1 month after discharge from hospital (T2)		
**Rodriguez, 2023**						
**Schmid, 2024**	Baseline (during outpatient visit before randomisation)	Immediately after the intervention (T1)<br><br>Directly before surgery and prior to any anaesthetic pre-meds (T2) *<br><br>**Preferentially selected*				
**Shao, 2019**	Before Intervention (T1)	1 hr before surgery (T2)	24hrs after surgery (T3)			
**Turrado, 2021**						
**Waller, 2022**	Baseline	Day before surgery				
**Rehabilitation Programs**						
**Alves, 2024**	Admission		Postoperative Day 2 *<br><br>Postoperative Day 7<br><br>**Preferentially selected*			

**Liu, 2024**	Before intervention initiation (M0)			1 month post intervention initiation (M1)	3 month post intervention initiation (M3)	6 month post intervention initiation (M6)
**Lv, 2024**	Preoperatively		1 week after discharge	1 month after surgery		
**Schrempf, 2023**	Admission		Postoperative Day 7	Postoperative Day 30		
**Yu, 2022**						6 months post discharge
**Both Prehabilitation and Rehabilitation**						
**Low, 2023**		Preoperatively	During inpatient recovery	Approximately 30 days after postoperative discharge		
**Min, 2024**						
**Schrempf, 2022**						
**Yuan, 2023**				Postoperatively, during the patients' hospitalisation period		

Table S6 – Prioritisation of timepoints:

As multiple timepoints were reported within the same domain, a predefined hierarchical approach was developed prior to data analysis to determine which timepoint was included in the pooled analyses. The timepoint most relevant to the predefined definition was preferentially selected. A detailed description of these selections is provided in the table below.

Table S6: Hierarchical prioritisation of assessment timepoints used in the included studies.

Timepoint Label	Prioritisation Hierarchy
**Baseline**	**Not required*
**Preoperative**	Timepoint closest to surgery (Schmid, 2024).
**Immediate Postoperative Period**	Timepoint closest to surgery (Alves, 2024).
**Early Postoperative Period**	**Not required*
**Intermediate Postoperative Period**	**Not required*
**Long-Term Postoperative Period**	**Not required*

Material S1– Prioritisation of outcomes for analysis

Material S1 – Prioritisation of measurements:

A predefined hierarchical approach was developed prior to data analysis and applied to select outcomes for pooled analyses when multiple metrics were reported within the same domain. Length of stay and hospital readmission were not subject to this hierarchy as we extracted the index hospitalisation duration and readmission events, respectively. No hierarchy was required for pain, fatigue or distress.

<u>Postoperative Complications:</u>

In the postoperative complications domain, we applied the following hierarchy:

1. Most widely used classification system (e.g., Clavien–Dindo Grading System) over less commonly used systems (e.g., Comprehensive Complication Index) to maximise comparability across studies. (Schrempf, 2022; Schrempf, 2023)
2. Composite incidence capturing all complications over incidence for a specific complication type (e.g., pulmonary infection) (Min, 2024).
3. Overall composite incidence over stratified incidence by severity grade (Rodriguez, 2023).
4. Stratified incidence (e.g., major complications) if no composite incidence was reported (Lv, 2024; Schrempf, 2022; Schrempf, 2023).

<u>Health-related Quality of Life:</u>

In the quality of life domain, we applied the following hierarchy:

1. Cancer-specific instruments (eg. EORTC QLQ-C30) over generic instruments (eg. EQ-5D) (Liu, 2024)
2. Summary health-related quality of life scores over individual domain scores.
    a. If a summary score was not reported, but the full dataset was available, we calculated the score manually using the scoring manual (Yu, 2022).
    b. If the summary score was not available, we preferred broader scores (e.g., Global Health Status) over specific functional or symptom domains (Liu, 2024).
3. WHOQOL physical health domain over psychological, social, and environmental domains (Rocamora González, 2022).

Note: not all instruments or domains are listed as not all were reported in a way that required consideration in our hierarchy.

Anxiety:

In the anxiety domain, we preferred the most widely used classification systems (e.g., Hospital Anxiety and Depression Scale; Spielberger State-Trait Anxiety Inventory) over less commonly used instruments (e.g., Self-rating Anxiety Scale; Visual Analog Scale) to maximise comparability (Liu, 2024; Shao, 2019).

- Note: no included study reported both the Hospital Anxiety and Depression Scale and the Spielberger State-Trait Anxiety Inventory.

Note: not all instruments or domains are listed as not all were reported in a way that required consideration in our hierarchy.

Depression:

In the depression domain, we preferred the most widely used classification systems (e.g., Hospital Anxiety and Depression Scale) over less commonly used instruments (e.g., Self-rating Depression Scale) to maximise comparability (Liu, 2024).

Note: not all instruments or domains are listed as not all were reported in a way that required consideration in our hierarchy.

Supplementary Figures

Figure S1 – Postoperative Complication and Hospital Readmission

Author, year	Events / Sample Size<br>Intervention	 Control	Risk ratio (95% CI)	Weight (%)
**Postoperative Complications**				
Lv, 2024	1 / 68	1 / 68	1.00 (0.02 to 49.67)	1
Min, 2024	9 / 59	19 / 59	0.47 (0.23 to 0.96)	20
Rodriguez, 2023	22 / 35	21 / 39	1.17 (0.79 to 1.72)	62
Schrempf, 2023	5 / 31	5 / 31	1.00 (0.32 to 3.11)	8
Turrado, 2021	3 / 58	4 / 68	0.88 (0.21 to 3.77)	5
Schrempf, 2022	3 / 18	2 / 18	1.50 (0.28 to 7.93)	4
Pooled effect (I^2=0%)			**0.95 (0.69 to 1.32)**	**100**
**Hospital Readmissions**				
Low, 2023	5 / 12	4 / 12	1.25 (0.44 to 3.55)	82
Lv, 2024	3 / 68	1 / 68	3.00 (0.32 to 28.13)	18
Pooled effect (I^2=0%)			**1.46 (0.57 to 3.76)**	**100**

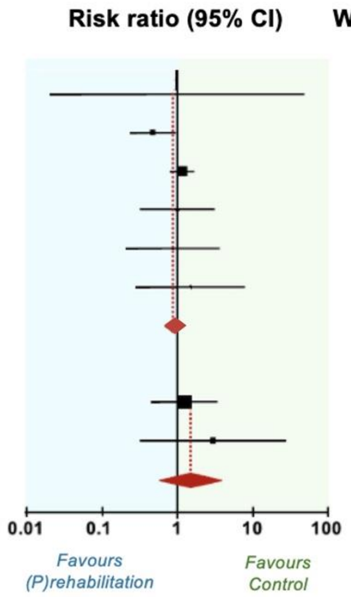

Figure S1: Risk of postoperative complications and hospital readmission. Risk ratios <1 favours prehabilitation interventions.

Figure S2 – Hospital Length of Stay

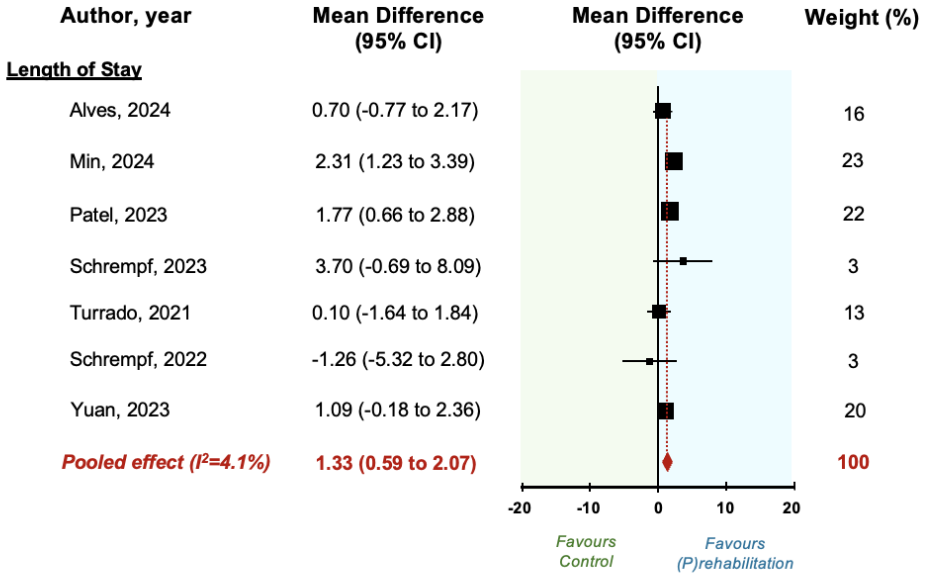

Figure S2: Mean difference for postoperative length of hospital stay (days) in randomised controlled trials of technology-enabled (p)rehabilitation for patients undergoing thoracic and/or abdominopelvic cancer surgery. Positive values favour prehabilitation interventions.

Figure S3 – Health-Related Quality of Life

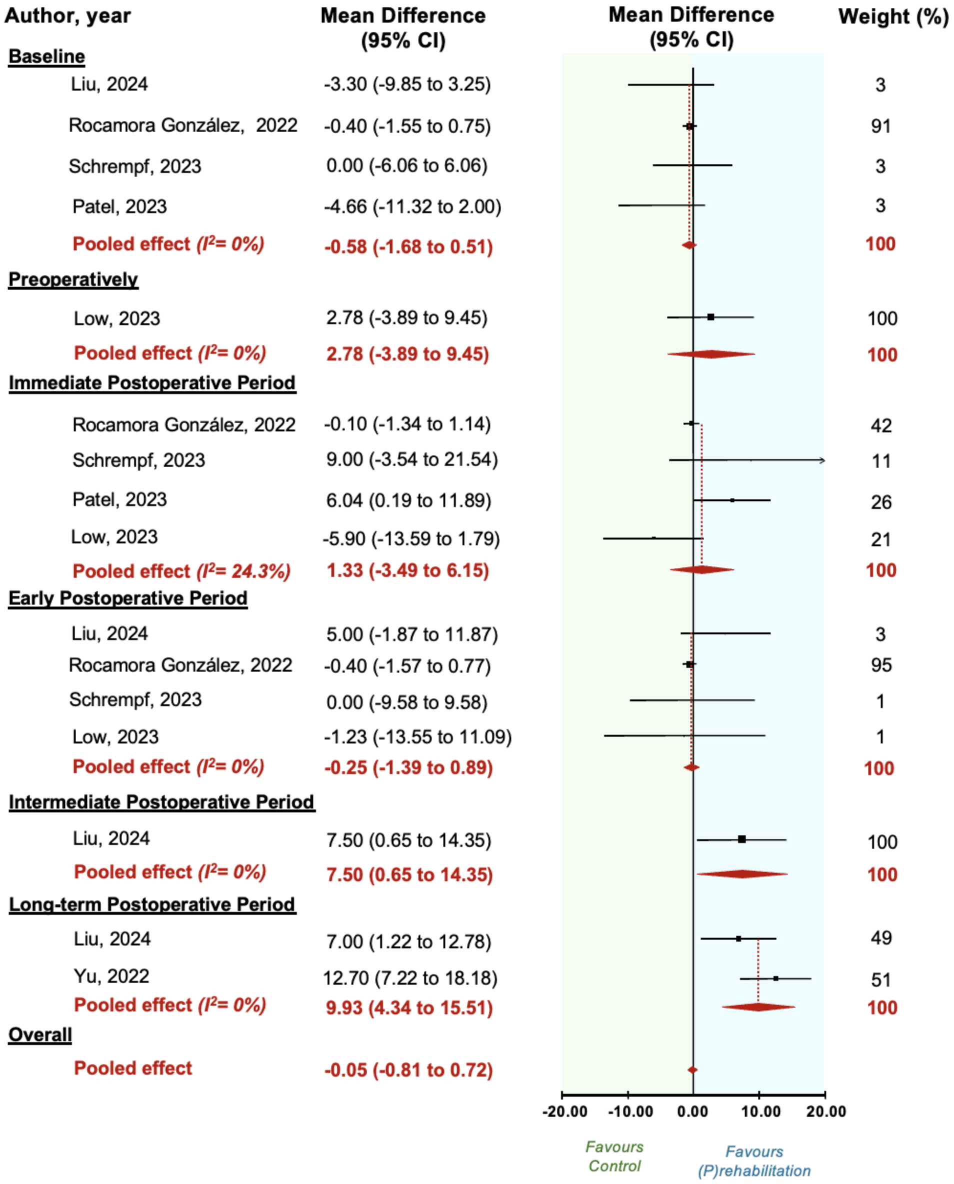

Figure S3: Mean difference in health-related quality of life in randomised controlled trials of technology-enabled (p)rehabilitation for patients undergoing thoracic and/or abdominopelvic cancer surgery. Positive values favour prehabilitation interventions.

Figure S4 – Pain

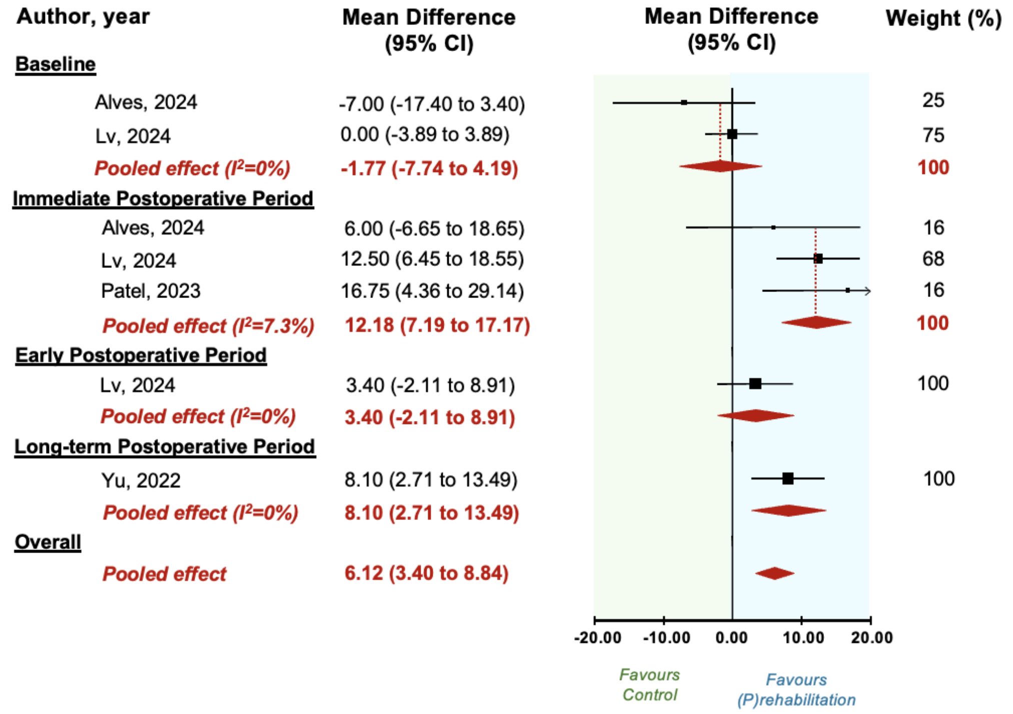

Figure S4: Mean difference in pain in randomised controlled trials of technology-enabled (p)rehabilitation for patients undergoing thoracic and/or abdominopelvic cancer surgery. Positive values favour prehabilitation interventions.

Figure S5 – Anxiety

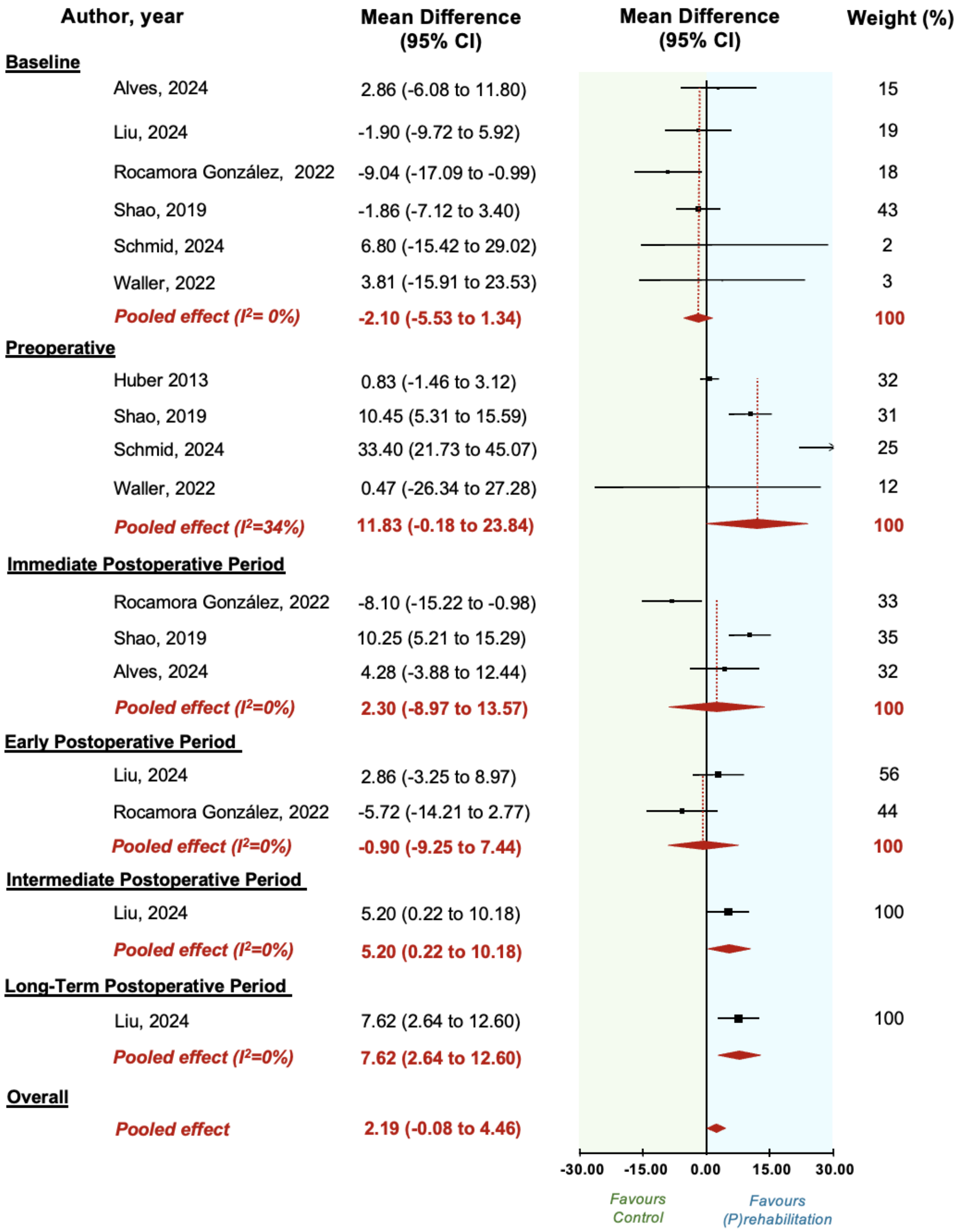

Figure S5: Mean difference in anxiety in randomised controlled trials of technology-enabled (p)rehabilitation for patients undergoing thoracic and/or abdominopelvic cancer surgery. Positive values favour prehabilitation interventions.

Figure S6 – Fatigue

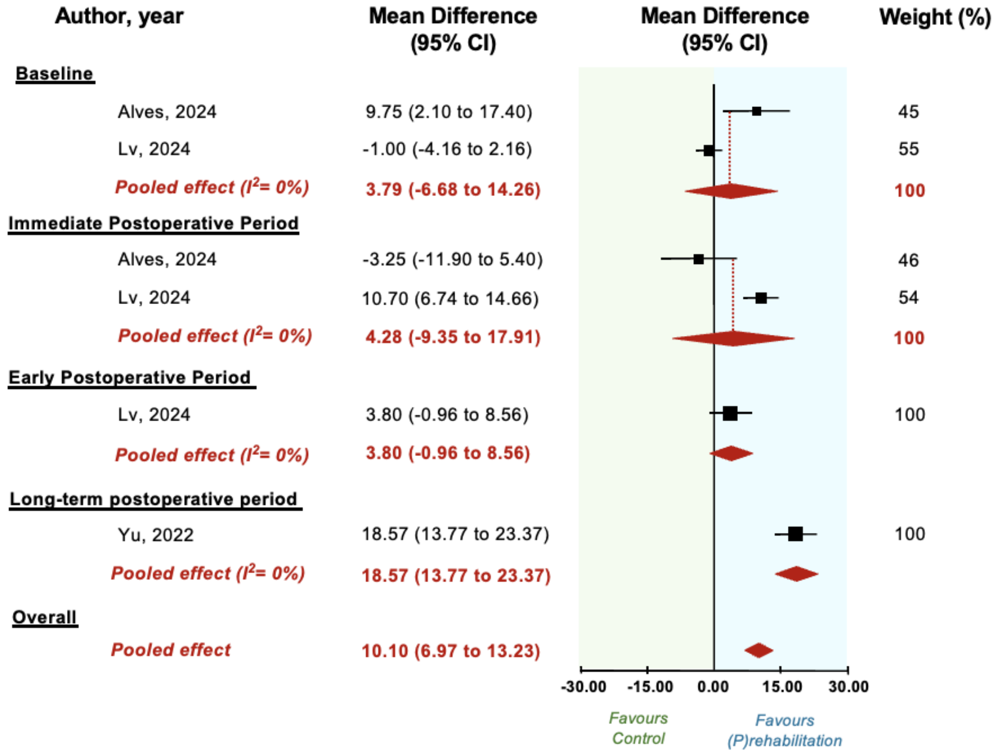

Figure S6: Mean difference in fatigue in randomised controlled trials of technology-enabled (p)rehabilitation for patients undergoing thoracic and/or abdominopelvic cancer surgery. Positive values favour prehabilitation interventions.

Figure S7 – Distress

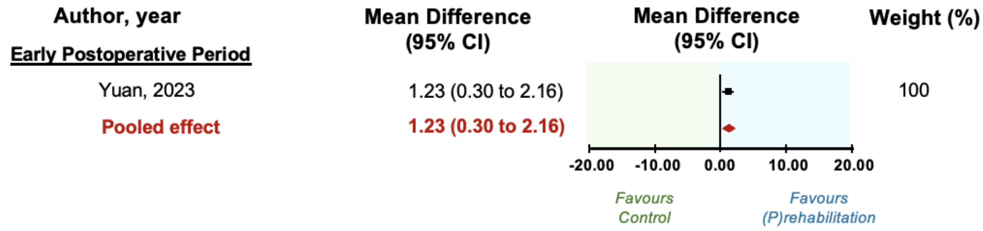

Figure S7: Mean difference in distress in randomised controlled trials of technology-enabled (p)rehabilitation for patients undergoing thoracic and/or abdominopelvic cancer surgery. Positive values favour prehabilitation interventions.

Table S7 – Patient Satisfaction

Table S7: Patient satisfaction outcomes reported in the included studies, stratified by program type.

Authors, Year	Tool / Measurement	Assessment Timepoints	Reported Satisfaction Outcome		Sample (n)	
			Intervention	Control	Intervention	Control
**Prehabilitation Programs**						
**Huber, 2013**	Self-designed questionnaire *(overall satisfaction; 1 = +++ , 6 = – – –)*	Preoperatively (6-10 hours after intervention delivery)	Mean = 1.4<br>SD = 0.6	Mean = 1.6<br>SD = 0.8	102	101
**Rocamora González, 2022**	Client Satisfaction Questionnaire (CSQ-8) *	End of study	Mean = 3.4 / 4<br>SD = not reported	N/A	Not reported	N/A
**Waller, 2022**	End of Study Questionnaire* *(overall program rating)*	End of study	Agreement Ratings:<br>Excellent = 5 (45%)<br>Good = 6 (55%)	N/A	11	N/A
**Rehabilitation Programs**						
**Lv, 2024**	Self-designed questionnaire * *(overall satisfaction; 0 = not satisfied, 5 = very satisfied)*	1 month after app use	95.5% (63/66) scored ≥ 3	N/A	66	N/A
**Schrempf, 2023**	European Organisation for Research and Treatment of Cancer (EORTC) inpatient satisfaction questionnaire *(overall patient satisfaction)*	Discharge	72.6	68.3	Not reported	Not reported
**Yu, 2022**	Likert scale *(1 = very dissatisfied; 5 = very satisfied)*	6 months after discharge	Mean = 4.80<br>SD = 0.64	Mean = 4.62<br>SD = 0.71	Not reported	Not reported
**Both Prehabilitation and Rehabilitation**						
**Low, 2023**	End-of-study interview *(overall satisfaction)*	End-of-study	Mean = 91.3<br>SD = 5.5	Mean = 86.2<br>SD = 11.0	9	11
**Schrempf, 2022**	Study specific questionnaire **	Discharge	No difference between the three groups (p=0.88)		Not reported	Not reported
**Yuan, 2023**	Nursing Satisfaction Score	Day before discharge	Mean = 98.32<br>SD = 1.66	Mean = 94.48<br>SD = 2.64	100	100

* Intervention group only

** Reported for the intervention and both control group
